# Universality in RNA and DNA deformations induced by salt, temperature change, stretching force, and protein binding

**DOI:** 10.1073/pnas.2218425120

**Published:** 2023-05-08

**Authors:** Fu-Jia Tian, Chen Zhang, Erchi Zhou, Hai-Long Dong, Zhi-Jie Tan, Xing-Hua Zhang, Liang Dai

**Affiliations:** ^a^Hubei Key Laboratory of Cell Homeostasis, College of Life Sciences, Renmin Hospital of Wuhan University, Wuhan University, Wuhan 430072, China; ^b^Department of Physics, City University of Hong Kong, Hong Kong 999077, China; ^c^Shenzhen Research Institute, City University of Hong Kong, Shenzhen 518057, China; ^d^School of Physics and Technology, Wuhan University, Wuhan 430072, China

**Keywords:** RNA structure, DNA structure, nucleic acid deformation, single-molecule experiment, molecular dynamics simulation

## Abstract

Nucleic acid deformations play important roles in many biological processes. The physical understanding of nucleic acid deformation by environmental stimuli is limited due to the challenge in the precise measurement of RNA and DNA deformations and the complexity of interactions in RNA and DNA. Magnetic tweezers experiments provide an excellent opportunity to precisely measure DNA and RNA twist changes induced by environmental stimuli. In this work, we applied magnetic tweezers to measure double-stranded RNA twist changes induced by salt and temperature changes. We observed RNA unwinds when lowering salt concentration, or increasing temperature. Our molecular dynamics simulations revealed the mechanism: lowering salt concentration or increasing temperature enlarges RNA major groove width, which causes twist decrease through twist-groove coupling. Combining these results with previous results, we found some universality in RNA and DNA deformations induced by three different stimuli: salt change, temperature, and stretching force. For RNA, these stimuli first modify the major groove width, which is transduced into twist change through twist-groove coupling. For DNA, these stimuli first modify diameter, which is transduced into twist change through twist-diameter coupling. Twist-groove coupling and twist-diameter coupling appear to be utilized by protein binding to reduce DNA and RNA deformation energy cost upon protein binding.

## Significance

DNA and RNA deformations play crucial roles in biological processes, such as DNA packaging and nucleic acid recognition by proteins. The relevant understanding is limited due to the challenge in the precise measurement of nucleic acid deformations and the complexity of nucleic acid interactions. We solve these two issues using experiments, simulations, and theory. Magnetic tweezers experiments provide an excellent opportunity to precisely measure DNA and RNA twist changes induced by salt, temperature change, and stretching. Surprisingly, our simulations and theory find that common deformation pathways drive DNA and RNA deformations induced by different stimuli. Furthermore, the common deformation pathways appear to be utilized by protein binding to reduce the energy cost of DNA and RNA deformations.

Double-stranded (ds) DNA and dsRNA deformations play crucial roles in many biological processes, such as DNA packaging (1–6), nucleic acid–protein interactions (7–10), and gene expression (11–13). The deformations can be induced by various stimuli, such as salt changes (14–20), temperature changes (21, 22), external forces (23–25), and protein binding (26). DsDNA and dsRNA deformations can exhibit in numerous forms, such as bending, twisting, diameter variation, and groove width variation. For instance, DNA sharp bending occurs in nucleosomes for DNA compaction (27). Twisting dsDNA by topoisomerases leads to supercoiling and facilitates DNA compaction in bacteria (28). Furthermore, undertwisting dsDNA lowers the energetic cost of separating the two strands and hence promotes DNA replication and transcription (29, 30). Conversely, overtwisting dsDNA enhances the separation energy cost and double-helix stability, which has been adopted by bacteria living at high temperatures (31).

Nucleic acid deformations strongly affect nucleic acid–protein binding affinity because proteins often recognize nucleic acids through shape readout (32–36). Upon protein binding, dsRNA and dsDNA usually deform from their standalone conformations (intrinsic shapes) to the conformations that best fit protein shapes and facilitate nucleic acid–protein binding attractions, such as hydrogen bond formation. A recent study demonstrated that a higher deformation energy cost weakens the dsRNA/dsDNA–protein binding affinity because the deformation energy offsets the binding attraction (36). They found that some DNA mismatches can substantially enhance protein–DNA binding affinities because the new DNA shapes with mismatches can better fit the protein–DNA binding geometry and reduce DNA deformation energy cost (36).

An intriguing property of nucleic acid deformations is that many structural parameters of nucleic acids are correlated, which means that the variation of one structural parameter causes the variation of another one. For instance, stretching DNA leads to overtwisting (23, 37), while stretching RNA leads to undertwisting (24). The correlations or couplings of structural parameters are not straightforward and are often counterintuitive. Extensive simulation and theoretical analysis have been carried out to reveal the mechanisms of these couplings (25, 38) as the couplings of nucleic acid structural parameters play essential roles in the responses of nucleic acid structures to environmental changes, such as salt and temperature changes (38–40).

While nucleic acid deformations are biologically important, the relevant understanding is limited because nucleic acid deformations are usually on very short lengths and the precise measurement of deformations is challenging. Some researchers used the DNA structures in the Protein Data Bank (PDB) to analyze DNA deformations induced by protein binding, while these DNA structures were precisely resolved by X-ray diffraction (36). However, the number of DNA structures in the database is limited. Furthermore, it is impossible or difficult to use these DNA structures to analyze the DNA deformations with environmental changes, such as salt and temperature. A high-throughput and precise method is demanded to measure nucleic acid deformations with environmental changes.

**Fig. 1.**
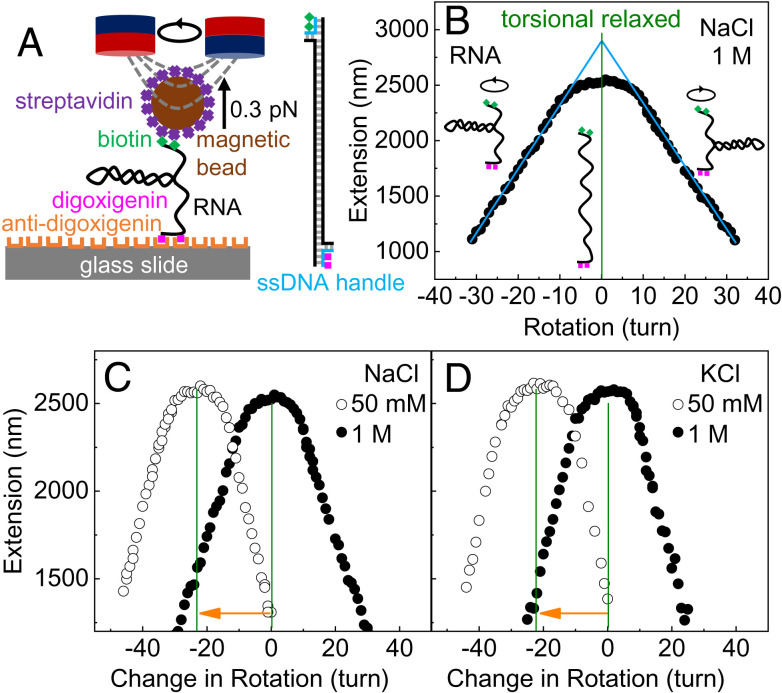
Experimental measurement of salt-induced twist changes of RNA. (*A*) The torsion-constrained RNA was attached between a glass slide and a superparamagnetic microbead through multiple digoxigenin and biotin groups. A pair of permanent magnets was used to stretch and rotate the RNA. (*B*) A representative bell-shape rotation–extension curve of RNA at 0.3 pN and 1 M NaCl. The torsion-relaxed point (vertical line) was determined as the position where the two linear fits at the positive and negative plectoneme regions (cyan lines) meet. (*C* and *D*) At each salt concentration, the shift in torsion-relaxed points relative to that at 1 M salt was measured.

In this work, we employed magnetic tweezers (MT) experiments to precisely measure RNA twist changes induced by salt and temperature changes. MT experiments provide an excellent opportunity to precisely measure DNA or RNA twist changes because even small twist changes can accumulate along a long DNA or RNA molecule and cause a large rotation of the DNA or RNA end (Fig. 1*A*), which has been demonstrated by recent studies using magnetic or optical tweezers (22–24, 37, 39–43). To explain the experimental results of salt- and temperature-induced RNA twist changes, we performed simulations and theoretical analysis. We found that RNA deformations induced by different environmental stimuli were driven by a common deformation pathway. Inspired by this, we carried out a similar analysis of DNA and also elaborated a common deformation pathway in DNA.

Our work aims to reveal the common deformation pathways in eight different phenomena: RNA and DNA deformations induced by salt, temperature change, stretching force, and protein binding. Among these eight phenomena, RNA deformations induced by salt and temperature changes are interesting results from our experiments and simulations. DNA and RNA deformations induced by protein binding are analyses of existing structures of protein–RNA/DNA complexes. RNA deformations by stretching force (24, 25, 38) as well as DNA deformations by stretching force (23, 25, 37, 38), salt (20, 39, 40), and temperature change (22, 40) have been measured by others and us and were remeasured here using additional DNA/RNA sequences.

## Results

### Salt-Induced RNA Twist Change.

We used MT experiments to measure the RNA twist changes with the salt concentration, *c*_salt_ (Fig. 1*A*) (16). At each *c*_salt_, we rotated a single torsionally constrained RNA and measured the RNA extension simultaneously, which yields the rotation–extension curves (Fig. 1*B*). We determined the torsionally relaxed point of RNA to be the crossing point of the two linear fits of the plectoneme ranges. Then, using the same RNA molecule, we changed *c*_salt_ and measured the torsionally relaxed point again (Fig. 1 *C* and *D*). We recorded the shift in the rotation turns *N*_turn_ induced by the salt change. Then, we converted *N*_turn_ to the twist change *Δω*_exp_ per base pair using *Δω*_exp_ = (*N*_turn_ × 360^*o*^)/*N*_bp_, where *N*_bp_ = 13.6 × 10^3^ is the number of base pairs. For convenience, we treated 1 M as the reference salt concentration, i.e., the RNA twist change *Δω*_exp_ = 0 at *c*_salt_ = 1 M.

In the above measurement, the change in *c*_salt_ slightly modifies the refractive index of the buffer, which slightly affects the measurement of RNA extension. As described above, determining salt-induced twist changes needs just the peak locations of the curves in Fig. 1 *C* and *D* but not the peak heights, i.e., the absolution values of RNA extension. The change in the refractive index has no influence on the peak locations and hence no influence on the results of RNA twist changes. For accuracy, the data of extensions in Fig. 1 have considered the effect of the change in the refractive index on RNA extension measurement (*SI Appendix*, section S2).

**Fig. 2.**
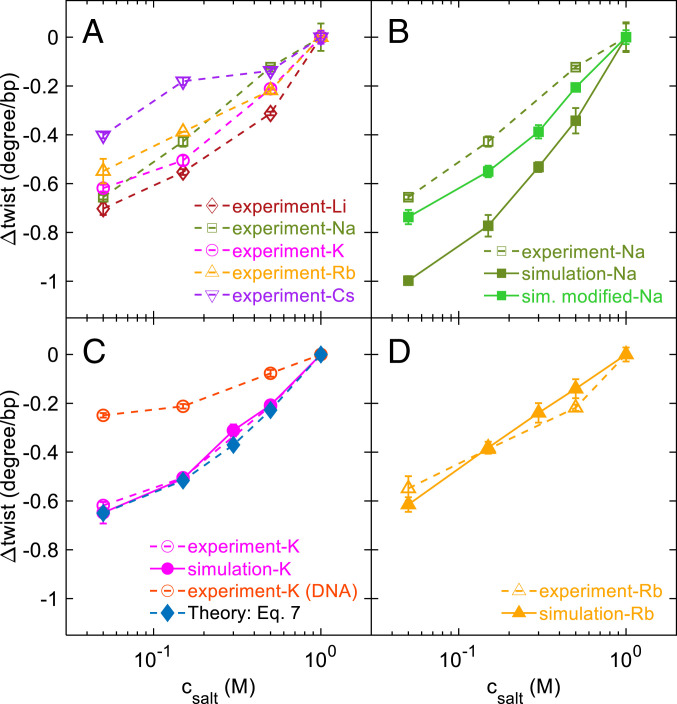
Twist angle as a function of the salt concentration measured by experiments and simulations. (*A*) Experimental results for LiCl, NaCl, KCl, RbCl, and CsCl. (*B*–*D*) Comparison of experimental results and simulation results for three salt species, Na^+^, K^+^, and Rb^+^. In (*B*), we add the simulation results using modified nonbonded parameters of Na^+^. In (*C*), we add the experimental results of DNA in KCl from our previous work (40) for comparison. Furthermore, we also add a theoretical prediction based on Eq. **7** with *α* = 1.8. The error bars for the MT data points are standard deviations obtained from at least three measurements and for the MD data points represent the standard deviations from the values of five equal intervals after equilibrium.

Fig. 2*A* presents the experimental results of RNA twist changes as a function of *c*_salt_ at the temperature of *T* = 295 K. With the decreasing of *c*_salt_, *Δω*_exp_ decreases steadily for all five monovalent ions. The salt-induced RNA twist changes are similar for all five ions, suggesting that the twist changes may be mainly caused by electrostatic screening.

In Fig. 2*A*, the magnitude of salt-induced RNA twist change over the range $0.05\;M\leq c_{\mathrm{salt}}\leq 1\;M$ follows the order: ${\mathrm{Cs}}^{+}<{\mathrm{Rb}}^{+}<K^{+}≲{\mathrm{Na}}^{+}<{\mathrm{Li}}^{+}$. The ion-type dependence may be caused by different binding patterns of different ions on RNA. In a recent similar study, Schwierz and coworkers investigated DNA twist changes induced by different cations (39). They pointed out that the cations binding on different sites of DNA have different or even opposite effects on DNA twist, and the binding pattern depends on the ion type. A similar reason may be responsible for the ion-type effect on RNA twist, which is an important topic for future studies.

In Fig. 2, we shifted *Δω* to zero at 1 M for all ions in order to observe the common trend. In addition, we shifted *Δω* because we did not measure the absolute RNA twist but measured the twist change in an RNA molecule when varying *c*_salt_. To obtain the RNA twist change induced by switching the ion type, we carried out a set of additional experiments where we measured the twist change in an RNA molecule when switching the ion type at a fixed *c*_salt_. We find that for a given *c*_salt_ between 0.05 M and 1 M, RNA twist roughly increases as ${\mathrm{Cs}}^{+}<{\mathrm{Rb}}^{+}<K^{+}<{\mathrm{Li}}^{+}<{\mathrm{Na}}^{+}$ (*SI Appendix*, section S3).

We also measured salt-induced RNA twist changes for two additional RNA sequences. In total, three RNA sequences were used and have GC contents of 36%, 43%, and 57%, respectively, while the data in Fig. 2 correspond to 43% GC. For these three RNA sequences, there are small but noticeable changes in the twist− < *SPSDOUBLEDOLLAR* > *c*_salt_ curves (*SI Appendix*, section S4). We did not observe a clear trend when varying the GC content. The sequence effect may be caused by different nucleic acid mechanical properties at different types of base pairs or base-pair steps (4, 44). For example, previous studies found that DNA pyrimidine–purine base-pair step has much greater twist flexibility than other types of base-pair steps (45). A similar trend was observed for RNA in our simulations: RNA sequences with higher fractions of pyrimidine–purine base-pair steps have greater twist flexibility and larger RNA twist changes induced by salt (*SI Appendix*, section S11).

### Semiquantitative Reproduction of Salt-Induced RNA Twist Change by Simulations.

To reveal the mechanism of the salt-induced RNA twist changes, we performed all-atom molecular dynamics (MD) simulations of 25-bp RNA at different concentrations of NaCl, KCl, and RbCl at *T* = 295 K. The sequence is CGACU CUACG GAAGG GCAUC UGCGC, which contains nine dinucleotide steps (46). For each *c*_salt_, we ran 600-ns MD simulation using GROMACS software (47) with OL3 force field (48). The trajectory of the last 500-ns simulation was used for data analysis. We calculated the H-twist of RNA using the Curves+ program (49). Only the helical parameters of central 19 bp of RNA were used for analysis to avoid the end effects.

As shown by the solid lines in Fig. 2*B*–*D*, the simulation results quantitatively agree with the measured experimental results in dashed lines. There is a noticeable deviation between the simulation and experimental results for ${\mathrm{Na}}^{+}$, which might be caused by the imperfection of OL3 force field or the incompatibility between RNA parameters and ${\mathrm{Na}}^{+}$ parameters. We also tried the ParmBsc0 force field in our simulations, and the results were similar to the OL3 force field. Inspired by the analysis in a recent study of salt-induced DNA twist changes by Schwierz et al. (39), we manually adjusted the parameters for ${\mathrm{Na}}^{+}$ to better fit experimental results. As shown in Fig. 2*B*, increasing the sigma value of ${\mathrm{Na}}^{+}$ (corresponding to ion radii) from 0.244 nm to 0.29 nm can improve the fit between simulations and experiments (*SI Appendix*, section S14). We hope such an attempt of reparameterization can help future refinement of the relevant force fields.

Semiquantitative reproduction of salt-induced RNA twist changes by MD simulations suggests that our simulations should capture the key features during RNA deformations. Then, we analyzed the structural changes of RNA in MD simulations and eventually deciphered the mechanism of the twist changes: The decrease of *c*_salt_ strengthens electrostatic repulsion between phosphate groups and hence widens the major grooves, which is transduced to the twist decrease through the twist-groove coupling.

### RNA Twist Change Driven by Twist-Groove Coupling.

In the following parts, we first elaborate the twist-groove coupling in RNA and then derive the salt-induced RNA twist changes using this coupling. The twist-groove coupling can be extracted from a two-dimensional potential of mean force (PMF) over the twist, *ω* and the major groove width, *G*:

[1] $$\begin{array}{c} P_{\text{sim}}\left(\omega ,G\right)=-\left(k_{B}T\right)\ln\left[\Omega \left(\omega ,G\right)\right], \end{array}$$

where *k*_B_ is the Boltzmann constant, *T* is the temperature, and *Ω*(*ω*, *G*) is the relative density of RNA conformations at a given *ω* and *G*.

**Fig. 3.**
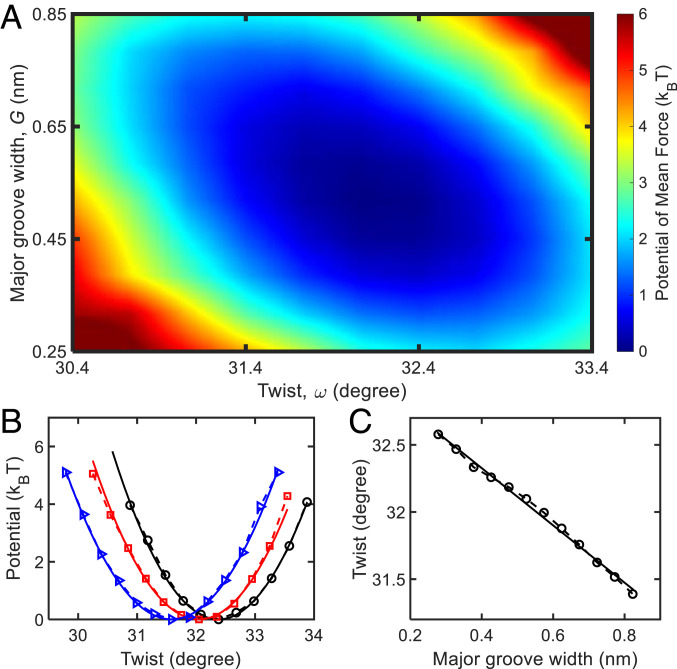
Free-energy analysis of the simulation result at 1 M KCl. (*A*) The two-dimensional potential of mean force (PMF) with respect to twist and major groove width of RNA from the simulation. (*B*) The one-dimensional PMF with respect to RNA twist for a narrow region of major groove width. The black, red, and blue symbols correspond to three regions: 0.3 < *G* < 0.4 nm, 0.5 < *G* < 0.6 nm, and 0.7 < *G* < 0.8 nm, respectively. The solid lines are quadratic fits: *y* = 1.8(*x* − 32.38)^2^, *y* = 1.7(*x* − 32.05)^2^, and *y* = 1.6(*x* − 31.59)^2^, respectively. (*C*) The average twist angle as a function of the major groove width. The symbols are from our simulation, and the solid line is a linear fit: *y* = −2.13(*x* − 33.18)^2^.

Fig. 3*A* presents the PMF from MD simulation at 1 M KCl, with a global minimum at *ω*_0_ = 32.15^*o*^ and *G*_0_ = 0.52 nm. *SI Appendix*, section S6 for the details of the calculation of *Ω*(*ω*, *G*). From the PMF, a negative coupling between *ω* and *G* is ascertained by the blue valley in Fig. 3*A*, which extends from the *Left Top* to the *Right Bottom*. The twist-groove coupling constant $k_{\omega G}^{\mathrm{bp}}$ can be determined by the fit of the simulation PMF to the following equation (23, 25, 40):

[2] $$\begin{array}{c} P^{\mathrm{bp}}\approx \frac{1}{2}k_{\omega }^{\mathrm{bp}}{\left(\Delta \omega \right)}^{2}+\frac{1}{2}k_{G}^{\mathrm{bp}}{\left(\Delta G\right)}^{2}+k_{\omega G}^{\mathrm{bp}}\Delta \omega \Delta G, \end{array}$$

whereas here $k_{\omega }^{\mathrm{bp}}$ and $k_{G}^{\mathrm{bp}}$ represent the twist rigidity and major groove width modulus, respectively. The superscripts of “bp” indicate that the coefficients correspond to the energies of one base pair. The fit of the data in Fig. 3*A* to Eq. **2** yields:

[3] $$\begin{array}{c} \begin{array}{cc} k_{\omega }^{\mathrm{bp}} & \approx 0.18\pm 0.01\;k_{B}T/\deg^{2},\\ k_{G}^{\mathrm{bp}} & \approx 3.61\pm 0.28\;k_{B}T/{\mathrm{nm}}^{2},\\ k_{\omega G}^{\mathrm{bp}} & \approx 0.43\pm 0.05\;k_{B}T/\left(\deg\cdot \mathrm{nm}\right). \end{array} \end{array}$$

The fitting quality can be seen in Fig. 3*B*. For a given *G*, the PMF can be approximated by a harmonic potential, and the location of the potential minimum shifts toward a smaller *ω* with the increase of *G*. The $k_{\omega }^{\mathrm{bp}}$ in Eq. **3** corresponds to an RNA twist rigidity of 589 pN ⋅ nm^2^, in agreement with experimental and simulation results (25, 38) (*SI Appendix*, section S8 for details). Meanwhile, $k_{\omega }^{\mathrm{bp}}$, $k_{G}^{\mathrm{bp}}$ and $k_{\omega G}^{\mathrm{bp}}$ are also calculated for other concentrations of KCl and other salt species, NaCl and RbCl (*SI Appendix*, Table S3 in section S7). We find that all three coefficients are insensitive to the salt concentration or species (*SI Appendix*, Fig. S6).

### Reproduction of Salt-Induced RNA Twist Change by Theoretical Calculation.

After obtaining the twist-groove coupling constant, we move to derive the twist changes based on this coupling constant. The derivation is based on the idea that the decrease in *c*_salt_ produces an effective force *f*_*G*_^salt^ to widen the major groove width, which reduces the twist through the twist-groove coupling. The *f*_*G*_^salt^ comes from the phosphate–phosphate (P–P) electrostatic repulsion and is estimated using the Debye screening formula:

[4] $$\begin{array}{c} f_{G}^{\text{salt}}=\frac{e^{2}}{4\pi \epsilon_{r}\epsilon_{0}}e^{-\frac{r}{\lambda_{D}}}\left[\frac{1}{r^{2}}+\frac{1}{r}\frac{1}{\lambda_{D}}\right]. \end{array}$$

where *r* is the P–P distance, *e* = 1.6 × 10^−19^ C is the unit charge, *ϵ*_0_ is the vacuum permittivity, *ϵ*_*r*_ ≈ 78.4 is the dielectric constant of water, and $\lambda_{D}=0.3034\;\mathrm{nm}/\sqrt{c_{\text{salt}}\left(\text{in}M\right)}$ is the Debye screening length. The above equation is derived from the derivative of the screened charge interaction potential $\frac{e^{2}}{4\pi \epsilon_{r}\epsilon_{0}}e^{-\frac{r}{\lambda_{D}}}$ with respect to *r*. For simplicity, we set *r* = 0.6 nm.

To derive how *f*_*G*_ is transduced to *Δω*, we add a term in Eq. **2**:

[5] $$\begin{array}{cc} P\left(c_{\text{salt}}\right)\approx & \;\frac{1}{2}k_{\omega }^{\mathrm{bp}}{\left(\Delta \omega \right)}^{2}+\frac{1}{2}k_{G}^{\mathrm{bp}}{\left(\Delta G\right)}^{2}\\ & +k_{\omega G}^{\mathrm{bp}}\Delta \omega \Delta G-\Delta f_{G}^{\text{salt}}\Delta G. \end{array}$$

Here, *Δf*_*G*_^salt^(*c*_salt_) ≈ *f*_*G*_^salt^(*c*_salt_) − *f*_*G*_^salt^(1M). *Δf*_*G*_^salt^ is the change in *f*_*G*_^salt^ when *c*_salt_ deviates from 1 M. Minimizing *P*(*c*_salt_) with respect to *Δω* and *ΔG* yields

[6] $$\begin{array}{c} \Delta \omega =\frac{-k_{\mathrm{wG}}^{\mathrm{bp}}}{k_{w}^{\mathrm{bp}}k_{G}^{\mathrm{bp}}-{\left(k_{\mathrm{wG}}^{\mathrm{bp}}\right)}^{2}}\Delta f_{G}^{\text{salt}}. \end{array}$$

**Fig. 4.**
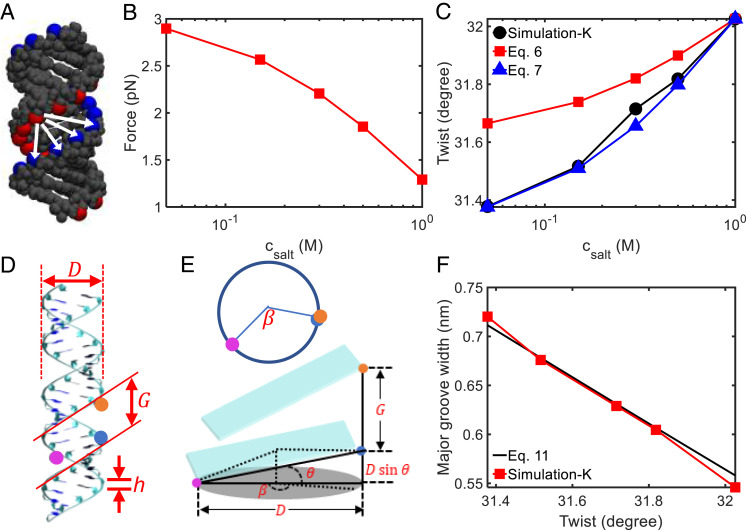
Salt-induced RNA twist change mediated by major groove width. (*A*) Illustration of phosphate group interactions in RNA. (*B*) Electrostatic force as a function of salt concentration. (*C*) Comparison of RNA twist changes from MD simulations of KCl and from Eqs. **6** and **7**. (*D* and *E*) Illustration of the molecular mechanism for twist-groove coupling. (*F*) Comparison of RNA major groove width versus twist from MD simulations of KCl and from Eq. **11**.

As shown in Fig. 4*C*, the prediction from Eq. **6** underestimates the twist changes. To achieve a better agreement, a rescaling factor *α* is added to *Δf*_*G*_^salt^:

[7] $$\begin{array}{c} \Delta \omega =\frac{-k_{\mathrm{wG}}^{\mathrm{bp}}}{k_{w}^{\mathrm{bp}}k_{G}^{\mathrm{bp}}-{\left(k_{\mathrm{wG}}^{\mathrm{bp}}\right)}^{2}}\left(\alpha \Delta f_{G}^{\mathrm{salt}}\right). \end{array}$$

Such rescaling is reasonable considering that Eq. **4** accounts for only one pair of P–P repulsion. In reality, each phosphate experiences interactions with many phosphate groups across the major groove (Fig. 4*A*). A rescale factor of *α* = 1.8 yields an excellent agreement between Eq. **7** and simulation results (Fig. 4*C*). Recall that we used the P–P distance of *r* = 0.6 nm in the above calculation. Using other values of *r* also leads to good agreements with simulation results of *Δω* after adjusting the rescale factor (*SI Appendix*, section S9). We note that in Eq. **4**, *r* should depend on *c*_salt_, but considering such dependence dramatically complicates the calculation. Furthermore, rigorous calculations of *f*_*G*_^salt^ needs to consider much more pairs of P–P repulsions (Fig. 4*A*) and are rather complex. Here, we aim to provide a simplified estimation of *Δf*_*G*_^salt^ in order to obtain a clear physical picture of how *Δf*_*G*_^salt^ is transduced to *Δω*. Technically, we use the rescaling factor *α* to absorb all approximations.

In this subsection, we present a simple theoretical model based on the mechanism that salt-induced RNA twist change is mediated by the variation of major groove width. This mechanism is supported by ion distribution around RNA: Cations strongly prefer to accommodate in RNA major grooves, as observed by a recent simulation study by Cruz-León and Schwierz (50). The strong preference of cations in RNA major grooves is because the negatively charged phosphate oxygens of the backbone point toward the interior of the major groove, as pointed out by Cruz-León and Schwierz (50). The strong preference of cations in RNA major grooves leads to the consequence that the variation of major groove width induced by salt is much larger than the variations of other RNA structural parameters (*SI Appendix*, Table S8 and section S12). The above theoretical model does not consider different ion distributions (different screening abilities) for different ion types (*SI Appendix*, section S10), which is an important topic for future studies.

### Temperature-Induced RNA Twist Changes Are also Driven by Twist-Groove Coupling.

**Fig. 5.**
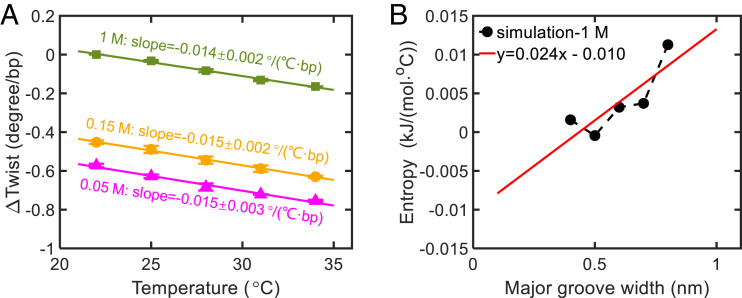
RNA twist change as a function of temperature. (*A*) MT measurements of RNA twist change with temperature for 0.05, 0.15, and 1 M KCl. *Δ*Twist is set to zero at 22 ° C and 1 M KCl. Linear fits to the experimental data yield the slopes as shown in the figure. (*B*) Dependence of RNA conformational entropy on major groove width extracted from MD simulations at 1 M KCl.

We also measured RNA twist change with temperature (Fig. 5*A*). We reveal the mechanism: At a higher temperature, RNA major groove width enlarges, which causes twist decrease through twist-groove coupling. Along with the mechanism, we derive equations for temperature-induced RNA twist changes. The derivation is similar as Eq. **6** except replacing *Δf*_*G*_^salt^ by *Δf*_*G*_^T^, the force of enlarging major groove width induced by temperature change. The calculation of *f*_*G*_^T^ is based on the following idea. The equilibrium major groove width, *G*, results from the competition of conformational entropy *S*(*G*), which tends to increase *G*, and the sum of interaction energies *U*(*G*), which tends to decrease *G*. The dependence of conformational entropy on *G* can be extracted from the PMFs of our MD simulations at different temperatures. The PMF can be expressed as *F*(*G*)=*U*(*G*)−*T* × *S*(*G*). After obtaining multiple sets of *F*(*G*) at different temperatures from simulations, we can solve *U*(*G*) and *S*(*G*) based on the approximation that *U*(*G*) and *S*(*G*) are insensitive to T. See the calculation details in *SI Appendix*, section S15. Fig. 5*B* shows the result of *S*(*G*) calculated using this method. From this result, we extract a coefficient, *k*_*SG*_, for the dependence of entropy on *G*:

[8] $$\begin{array}{c} k_{\mathrm{SG}}\equiv \frac{\partial S}{\partial G}\approx 0.024\;\mathrm{kJ}/\left(\mathrm{mol}\cdot {}^{{}^{\circ}}C\cdot \mathrm{nm}\right). \end{array}$$

The positive value of *k*_*SG*_ indicates that the conformational entropy increases with *G*, which supports our argument above: The conformational entropy tends to increase *G*. With the coefficient *k*_*SG*_, we obtain the force of enlarging major groove width induced by temperature change:

[9] $$\begin{array}{c} \Delta f_{G}^{T}\approx -\frac{\partial \Delta F}{\partial G}\approx \frac{\partial (S\Delta T)}{\partial G}\approx \Delta T\frac{\partial S}{\partial G}\approx k_{\mathrm{SG}}\times \Delta T. \end{array}$$

Substituting the above equation into Eq. **6**, we obtain the coefficient for temperature-induced RNA twist change:

[10] $$\begin{array}{c} \begin{array}{c} \frac{\Delta \omega_{\mathrm{bp}}}{\Delta T}\approx \frac{-k_{\omega G}^{\mathrm{bp}}}{k_{\omega }^{\mathrm{bp}}k_{G}^{\mathrm{bp}}-{\left(k_{\omega G}^{\mathrm{bp}}\right)}^{2}}k_{\mathrm{SG}}\approx -0.012^{{}^{\circ}}/\left({}^{{}^{\circ}}C\cdot \mathrm{bp}\right). \end{array} \end{array}$$

The above coefficient agrees with our experimental result, −0.014 ° /(°C ⋅ bp), as shown in Fig. 5*A*.

### The Molecular Mechanism Under RNA Twist-Groove Coupling.

It is interesting to find out the molecular mechanism for the twist-groove coupling. To establish a relationship between the major groove width and the twist, we make the following approximation. As shown in Fig. 4*D*, the major groove width corresponds to the distance between the orange and blue points (two phosphate sites). Note that the distance is along the vertical direction, i.e., the axis of the double-helix. We design another path from the orange point to the blue point: first walking from the orange point to the magenta point (the phosphate on the same backbone strand) and walking from the magenta to the blue point on the complementary strand. Both paths share the same vertical distance, i.e.,

[11] $$\begin{array}{c} G\approx hN_{\mathrm{bp}}^{\text{groove}}-D\sin\theta \approx h\frac{\beta }{\omega }-D\sin\theta , \end{array}$$

where *h* is the vertical distance between two adjacent base pairs (or the helical rise), $N_{\mathrm{bp}}^{\text{groove}}$ is the number of base pairs from the orange point to the magenta point, *D* = 1.95 nm is RNA diameter, *θ* = 15.5 ∼ 17.5° is the angle between the base-pair plane and the horizontal plane (or the inclination angle), *β* ≈ 140° is the azimuthal angle between two strands in RNA and *ω* is the twist angle per base-pair step. Eq. **11** uses $N_{\mathrm{bp}}^{\text{groove}}\approx \beta /\omega$, which is a key step relating *ω* to *G*. The prediction of Eq. **11** agrees well with the simulation results from 1 M KCl (Fig. 4*F*). The physical picture behind Eq. **11** is that the two phosphate groups across the major groove correspond to a roughly fixed rotation angle along the backbone strand, and a smaller twist angle leads to a larger number of rotation steps (base pair steps) and thus a larger groove width.

### Universality in RNA and DNA Deformations.

**Fig. 6.**
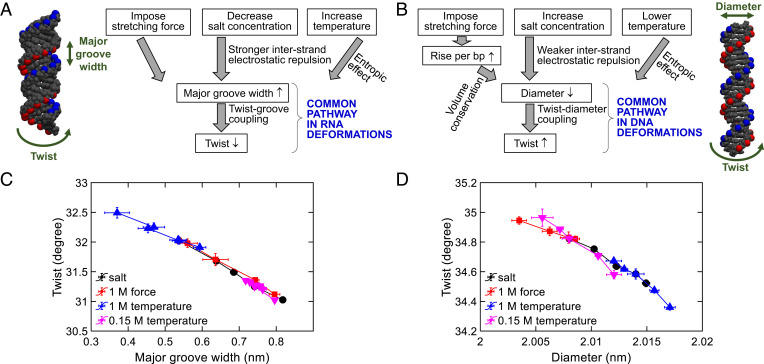
Common deformation pathways in RNA and DNA. (*A*) RNA twist changes induced by three stimuli are commonly mediated by twist-groove coupling. (*B*) DNA twist changes induced by three stimuli are commonly mediated by twist-diameter coupling. (*C*) Our simulation results of RNA twists versus major groove widths when varying salt, force, or temperature. (*D*) Our simulation results of DNA twists versus diameters when varying salt, force, or temperature.

Inspired by the above results as well as the results in previous studies (23, 25, 38, 40), we identified some universality in RNA and DNA deformations, as illustrated in Fig. 6 *A* and *B*. For RNA, stretch-, salt-, and temperature-induced twist changes are commonly mediated by twist-groove coupling, although the first step of the deformation pathway differs among these three stimuli. For DNA, stretch-, salt-, and temperature-induced twist changes are commonly mediated by twist-diameter coupling (23, 40).

Fig. 6*C* presents a scatter plot of RNA twist versus major groove width. Three curves collapse, which supports that RNA twist change is primarily mediated by twist-groove coupling. In addition, our theoretical equations quantitatively reproduce salt- and temperature-induced RNA twist changes based on capturing the variation of RNA major groove width. This reproduction also supports that RNA twist changes by force, salt, and temperature are primarily mediated by major groove width variation and twist-groove coupling.

Fig. 6*D* presents the results of DNA deformations. Three curves also collapse. In addition, theoretical equations can also quantitatively reproduce salt- and temperature-induced DNA twist changes based on capturing the variation of DNA diameter. These results support that DNA twist changes by force, salt, and temperature are primarily mediated by diameter variation and twist-diameter coupling.

### Difference Between RNA and DNA Deformations.

A natural question arises from the above results is that why RNA deformations are mediated by major groove width variation and twist-groove coupling, but DNA deformations are mediated by diameter variation and twist-diameter coupling. A simple answer is that RNA and DNA choose the deformation pathways with the lowest energy cost for themselves (25, 38). The extra oxygen of RNA with respect to DNA impedes RNA diameter variation (25).

In addition, our experiments observed that the magnitudes of salt-induced RNA twist changes are roughly two or three times of DNA (Fig. 2*C*). One possible reason is as follows. Salt-induced twist changes for both RNA and DNA are transduced through two steps: i) salt change to major groove width or diameter change mediated by electrostatic repulsions and ii) major groove width or diameter change to twist change mediated by the coupling of twist with groove or diameter. For DNA, the coefficients related to twist-diameter coupling are *k*_*ω*_ = 0.18 ± 0.02 *k*_B_*T*/deg^2^, *k*_*D*_ = 263 ± 39 *k*_B_*T*/nm^2^, *k*_*ωD*_ = 4.5 ± 0.8 *k*_B_*T*/(deg ⋅ nm), which were obtained from our previous study (40). For RNA, both two transduction steps appear to be strong, while for DNA, the first step is moderate, and only the second step is strong. Eventually, the effect of salt-induced twist changes is stronger for RNA. See more discussion in *SI Appendix*, section S12.

### The Two Couplings Facilitate Nucleic Acid–Protein Binding.

**Fig. 7.**
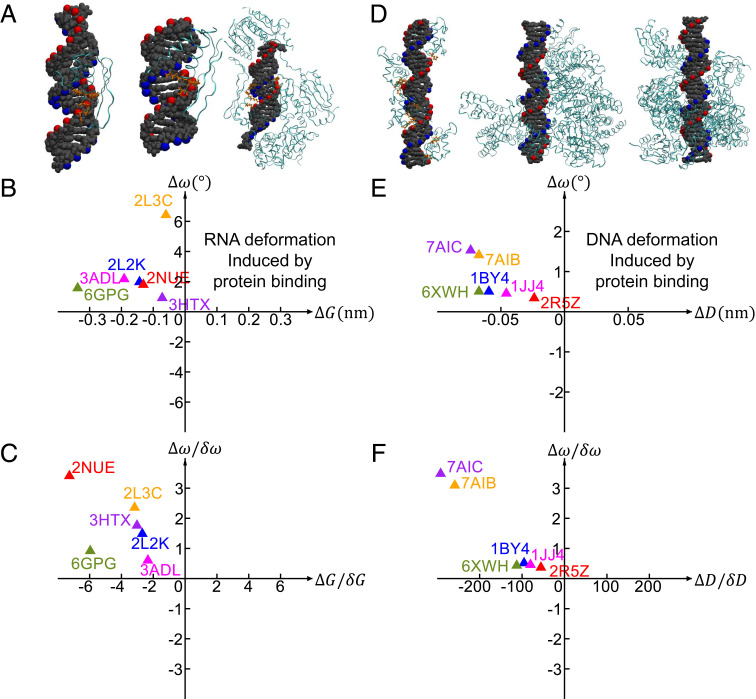
RNA and DNA deformations induced by protein binding. (*A*) Examples of RNA–protein complexes. (*B*) *Δω* and *ΔG* are the differences in the twist and major groove width between RNA–protein complexes with the corresponding standalone RNA structures, respectively. (*C*) Normalizing *Δω* and *ΔG* by *δω* and *δG*, where *δω* and *δG* are the standard deviations of *ω* and *G* under thermal fluctuations, respectively. (D–F) Similar results for DNA except *ΔG* are replaced by *ΔD*.

The twist-groove coupling of RNA may affect RNA–protein binding affinity. If the RNA deformation induced by protein binding is along the direction of the twist-groove coupling, then the coupling can reduce RNA deformation energy and facilitate RNA–protein binding. To examine this hypothesis, we analyzed the RNA deformations in six RNA–protein complexes: i) ADAR2 dsRBD2 (PDB: 2l2k) (51), ii) ADAR2 dsRBD1 (PDB: 2l3c) (51), iii) Plant small RNA methyltransferase HEN1 (PDB: 3htx) (52), iv) *A*. *aeolicus* RNase III (PDB: 2nue) (53), v) *H. sapiens* TRBP (PDB: 3adl) (54), and vi) RIG-I Singleton-Merten syndrome variant C268F (PDB: 6gpg) (55). The structures of the first three complexes are shown in Fig. 7*A*. We picked these RNA–protein complexes because in these complexes, proteins mainly bind on RNA major grooves, which are likely to vary RNA major groove widths. For each RNA–protein complex, we performed MD simulations for the standalone RNA and calculated *ω* and *G*. Then, we calculate *Δω* = *ω*_complex_ − *ω*_RNA_, where *ω*_complex_ and *ω*_RNA_ are the twists of the RNA–protein complex and standalone RNA, respectively. Similarly, we calculated *ΔG* = *G*_complex_ − *G*_RNA_. The scatter plot of *ΔG* and *Δω* in Fig. 7*B* indicates that the three sets of *ΔG* and *Δω* are along the direction of twist-groove coupling, which confirms our hypothesis. In addition, *ΔG* is negative, and *Δω* is positive. Accordingly, we speculate that protein binding may compress RNA major groove width, which is transduced into twist increase through twist-groove coupling. We notice that the data points in Fig. 7*B* are spreading. One possible reason is that RNA twist flexibility depends on sequence (45), which affects *Δω*. The twist flexibility can be reflected by *δω*, which is the SD of twist under thermal fluctuations. Fig. 7*C* presents the data points after normalizing *Δω* by *δω*. Similarly, we normalize *ΔG* by *δG*. Such normalizations appear to significantly reduce the spreading of the data points, suggesting that the intrinsic twist flexible and groove flexibility, which are sequence-dependent, play roles in RNA deformations.

We carried out similar analysis for six DNA–protein complexes as shown in Fig. 7 *D* and *E*. We picked these DNA–protein complexes because in these complexes, proteins mainly wrap around DNA, which are likely to vary DNA diameters. The three sets of *ΔD* and *Δω* are along the direction of twist-diameter coupling. For these three complexes, proteins wrap around DNA. In addition, *ΔD* is negative, and *Δω* is positive. Accordingly, we speculate that protein binding may compress DNA diameter, which is transduced into twist increase through twist-diameter coupling.

## Discussion

In summary, we precisely measured RNA twist changes with salt and temperature by leveraging the accumulation of small twist change along an RNA chain. The precise experimental results of RNA twist changes (precision ∼ 0.01 ° /bp) provide a unique window to probe nucleic acid deformation pathways. Combining the experiments with simulations and theoretical analysis, we revealed and quantified RNA twist-groove coupling in RNA, which is the common driving force for salt, temperature, and stretch-induced RNA twist changes. Similarly, DNA twist-diameter coupling is the common driving force for salt, temperature, and stretch-induced DNA twist changes. Very intriguingly, we find that RNA twist-groove coupling and DNA twist-diameter coupling appear to take effect in nucleic acid–protein binding—that is, nucleic acid deformations induced by protein binding are along the direction of RNA twist-groove coupling or DNA twist-diameter coupling. Identifying the common deformation pathway, i.e., RNA twist-groove coupling or DNA twist-diameter coupling, should provide a simplified and quantitative view of nucleic acid deformations on many occasions.

While this work focuses on the commonality of RNA and DNA deformations induced by different stimuli, the specificity, such as ion-type effect and sequence effect, also plays crucial roles in RNA and DNA deformations, which are important topics for further investigation. The ion-type effect on RNA twist is strongly linked to the ion binding and distribution. Accordingly, the experimental measurement of salt-induced RNA twist, combined with MD simulations, provides a unique window to probe ion distribution around RNA and to refine MD force fields, just as what has been done for DNA by Schwierz and coworkers recently (39). The sequence also plays many important roles in RNA and DNA deformations, such as affecting bending flexibility (4, 44), twist flexibility (45), and ion distributions (39). The sequence effects on the shapes and deformations of nucleic acids are involved in many biological functions, such as protein recognition of nucleic acids (33) and genome packaging (44).

## Materials and Methods

### Magnetic Tweezers Experiments.

We built the magnetic tweezers on an inverted microscope (IX73, Olympus) equipped with a 100x oil immersion objective (NA1.4). We labeled one end of the RNA/DNA with multiple digoxigenin groups and labeled the other end of the RNA/DNA with multiple biotin groups (19, 56) (*SI Appendix*, section S1). We attached the digoxigenin end of RNA/DNA to the inner bottom surface of the flow cell which was coated with antidigoxigenin (Roche). We attached the biotin end of RNA/DNA to a superparamagnetic microbead (Dynabeads^TM^ MyOne^TM^ Streptavidin C1). We used a pair of separated NdFeB magnets to apply constant forces to the magnetic bead and stretch the RNA/DNA. We rotated the RNA/DNA by rotating the magnets using a rotation motor (DT-34 DC, Physik Instrumente). We used a high-brightness collimated LED (MLS-201, Wuhan Yibo) to illuminate the flow cell through the gap between the magnets. We captured the images with a CCD camera (VC-12MC-M65, Vieworks) and analyzed the 3-D position of the beads based on the diffraction pattern of the beads. We used an objective heater (Bioptechs) to wrap around the oil objective to control the temperature in the flow cell.

With the above experimental setup, we measured RNA twist changes induced by salt change using a procedure similar to previous works (22, 57, 58). We fixed the applied force at 0.3 pN and rotated the RNA end using magnets. During the rotation, we recorded the RNA extension to obtain the rotation–extension curve as shown in (Fig. 1). In each step, we rotated one turn and stayed at the rotation angle for ten seconds. During the ten seconds, we recorded the RNA extension and calculated the average extension. Eventually, we obtained the rotation–extension curve after many times of rotations. Our purpose is to determine the torsionally relaxed point of RNA, which corresponds to the maximum extension. Note that around the maximum extension, the extension changes very slowly with the rotation, which makes the determination of the torsionally relaxed point not very precise. To improve the precision, we usually rotated ± 20 turns relative to the torsionally relaxed point and then determined the torsionally relaxed point as the crossing point of the two linear fits at the positive and negative plectoneme regions of the twist-extension curve (Fig. 1*B*). For each salt concentration of each ion type, we repeated the measurement using different RNA molecules in different flow cells for at least three times. The above procedure was also applied to measure temperature-induced RNA twist changes as well as salt- and temperature-induced DNA twist changes. *SI Appendix*, section S1 for more experimental details.

### Atomistic Molecular Dynamics Simulations.

We first constructed an A-form RNA duplex with the sequence of CGACU CUACG GAAGG GCAUC UGCGC (46) using 3DNA (59). The simulation system containing the dsRNA molecule was then filled with TIP3P water molecules and monovalent ions (${\mathrm{Na}}^{+}$, $K^{+}$, ${\mathrm{Rb}}^{+}$, or ${\mathrm{Cs}}^{+}$) at desired concentrations. A production run of 600 ns was carried out for each simulation system after energy minimization and equilibration. Our all-atom MD simulations used the GROMACS program (47) and OL3 force field (48). We used the Curves+ program to analyze RNA structures (49). *SI Appendix*, section S5 for more simulation details.

## Supplementary Material

Appendix 01 (PDF)

## Data Availability

The data and codes are available at https://github.com/cityuBiophysics/RNA-DNA-deformations (60). All study data are included in the article and/or *supporting information*.
