# Supplementary material for: Universality in RNA and DNA deformations induced by salt, temperature change, stretching force, and protein binding
Source: Proc Natl Acad Sci U S A. 2023 May 8;120(20):e2218425120. doi: 10.1073/pnas.2218425120 (PMC10193934; doi:10.1073/pnas.2218425120)
Supplement: Supplementary file 1 — Appendix 01 (PDF) [file pnas.2218425120.sapp.pdf]

PNAS

# Supporting Information for

## Universality in RNA and DNA Deformations Induced by Salt, Temperature Change, Stretching Force and Protein Binding

**Fu-Jia Tian, Chen Zhang, Erchi Zhou, Hai-Long Dong, Zhi-Jie Tan, Xing-Hua Zhang, and Liang Dai**

**Liang Dai and Xing-Hua Zhang.**
**E-mail: liangdai@cityu.edu.hk or zhxh@whu.edu.cn**

**This PDF file includes:**

Figs. S1 to S23
Tables S1 to S13
SI References

## Section S1. Details of single-molecule magnetic-tweezers experiments

**(I).** Temperature and solutions

We performed all single-molecule experiments in a cell culture room with a constant temperature of 22 °C. We prepared filtrated, autoclaved stocks of 4 M LiCl, NaCl, KCl, RbCl, and CsCl (Sigma-Aldrich). We purchased 1 M Tris-HCl pH 8.0 buffer from Sigma-Aldrich. Just before single-molecule experiments, we diluted the salt and Tris-HCl stocks in autoclaved DI water to specific salt concentrations.

**(II).** Torsion-constrained RNA

We prepared the torsion-constrained RNA using sequences from 13751 bp of lambda DNA containing 43.3% GC content, the same as the RNA used in our recent works (1, 2). Briefly, we firstly amplified the two multiple-labeled and short ssDNA strands either containing multiple biotin groups or digoxigenin groups by PCR with 30% biotin-11-dUTP (Roche) or digoxigenin-11-dUTP (Roche) . Then, we generated two long ssRNA strands using T7 RNA polymerase (New England Labs). We annealed above two ssRNA strands and two ssDNA strands together equimolarly through a process containing a one-hour incubation step at 65 °C followed by a one-hour slow cooling process from 65 to 30 °C. In practice, we found $\sim$ 40% of RNA molecules were successfully torsion-constrained.

**(III).** Flow cell

Briefly, we functionalized the Piranha-cleaned cover glass slides by 1% APTES (Sigma-Aldrich) - 2% glutaraldehyde (Sigma-Aldrich) - 0.1 mg/mL anti-digoxigenin (Roche), and passivated them using 200 mM Tris-HCl pH 8.0 (Sigma-Aldrich) and 2% BSA (Sigma-Aldrich). We pasted two double-sided adhesive tapes to the functionalized cover glass slide and then put a smaller cover glass slide on the top to form a flow cell. The flow cell has a capacity of $\sim$ 40 $\mu$L solution.

**(IV).** RNA and bead tethering

We diluted the RNA to $\sim$ 2 ng/mL in 10 mM Tris-HCl, 500 mM NaCl and anchored the RNA to the glass slide of the flow cell by 10-minutes of incubation. Then, we diluted the microbeads (M-270 streptavidin, Dynal) 100-fold and attached the beads to the free end of RNA after 10 minutes of incubation in the flow cell. The unbound beads are removed by excessively rinsing using about 10 mL 10 mM Tris-HCl pH 8.0.

**(V).** Torsion-extension curves measured by magnetic-tweezers

Briefly, we rotated the magnets one turn by one turn at a constant force of 0.3 pN. After each rotation turn, we recorded the extension in RNA for ten seconds and calculated the average in extension. Usually, we measured the extension in RNA in the range of ± 20 turns flanking the torsional relaxed point of the RNA, generating a bell-like torsion-extension curve. Then, we changed to another salt concentration and measured the torsion-extension curve using the same RNA molecule. At each salt concentration, we used at least three RNA molecules in different flow cells to calculate the twist as a function of salt concentration.

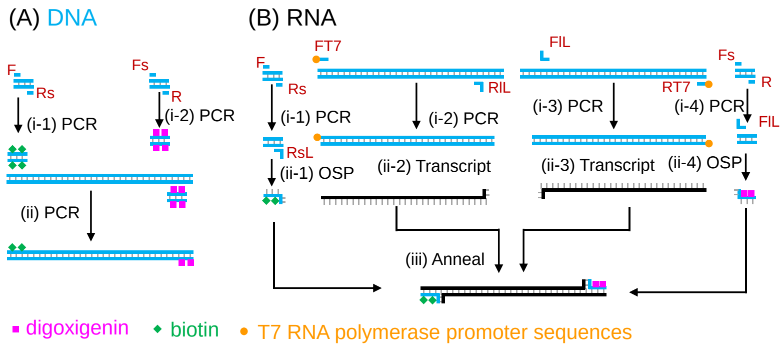


**Fig. S1.** Flowchart to prepare the torsion-constrained (A) DNA and (B) RNA constructs. All the PCR primers are indicated in red text.

**(VI).** Details of preparing torsion-constrained DNA constructs

(i-1) Prepare a multiple-biotin-labeled short DNA fragment by PCR using F and Rs as primers together with 30% biotin-11-dUTP (Thermo Fisher Scientific) and using lambda DNA as the template.

(i-2) Prepare a multiple-digoxigenin-labeled short DNA fragment by PCR using Fs and R as primers together with 30% digoxigenin-11-dUTP (Roche) and using lambda DNA as the template.

(ii) Prepare the torsion-constrained DNA construct by PCR using lambda DNA as the template and using the two short DNA fragments as mega-primers.

**(VII).** Details of preparing torsion-constrained RNA construct

(i-1) Make a short DNA template through PCR using F and Rs as primers and using lambda DNA as the template.

(ii-1) Then, generate the multiple-biotin-labeled ssDNA through one-sided PCR (OSP) using RsL as the primer together with 30% biotin-11-dUTP.

(i-2) Make a long DNA fragment through PCR using FT7 and RlL as primers and using the lambda DNA as the template.

(ii-2) Then, generate an ssRNA strand using T7 RNA polymerase.

(i-3) Make a long DNA fragment through PCR using F1L and RT7 as primers and using lambda DNA as the template.

(ii-3) Then, generate an ssRNA strand using T7 RNA polymerase.

(i-4) Make a short DNA fragment through PCR using Fs and R as primers and using lambda DNA as the template.

(ii-4) Then, generate the multiple-digoxigenin-labeled ssDNA through OSP using FsL as the primer together with 30% digoxigenin-11-dUTP.

(iii) Anneal above two ssRNA strands and two ssDNA strands together equimolar through a temperature process containing a one-hour incubation step at 65 °C followed by an over one-hour slow cooling process from 65 °C to 25 °C (-0.5 °C/min) without purification after OSP or transcription.

We used a set of primers for each sequence containing 43%, 57% or 36% GC percentage:

**Sequence 1, 43% GC, 24116-37683 of lambda DNA.** We used this DNA sequence for experiments in the main text.

*GC43_F: GCTTGGCTCTGCTAACACGTTGCTCATAGGAG*
*GC43_FT7: TAATACGACTCACTATAGGGCTCTGCTAACACGTTGCTCATAGGAG*
*GC43_R: AATTTAGCCCTTCAATCGCCAGAGAAATCTAC*
*GC43_RT7: TAATACGACTCACTATAGGGAATTTAGCCCTTCAATCGCCAGAGAAATCTAC*
*GC43_Rs: CAGCTACAGTCAGAATTTATTGAAGCAA*
*GC43_Fs: CCCTAAGACCTTTAATATATCGCCAAATAC*
*GC43_FsL: CATGCAATTATTGTGAGCAATACACACGCGCTTCCCCTAAGACCTTTAATATATCGCCA*
*GC43_RsL: CATGCAATTATTGTGAGCAATACACACGCGCTTCCAGCTACAGTCAGAATTTATTGAAG*
*GC43_R1L: TCATGCAATTATTGTGAGCAATACACACGCGCTTCGCAACAGATATTGAAGGGGAGC*
*GC43_F1L: TCATGCAATTATTGTGAGCAATACACGCGCTTCCTGAAACGTTGCGGTTGAACTAT*

**Sequence 2, 57% GC, 13657-21053 of lambda DNA.**

*GC57_F: CCCACGCTGACGGTTTCTAACC*
*GC57_FT7: TAATACGACTCACTATAGGGCCCACGCTGACGGTTTCTAACC*
*GC57_R: GTTTACCCGCAAGCGCGTTAG*
*GC57_RT7: TAATACGACTCACTATAGGGGTTTACCCGCAAGCGCGTTAG*
*GC57_Fs: CATCATCAAGTGCCGGTCGTGCAG*
*GC57_FsL: CATGCAATTATTGTGAGCAATACACACGCGCTTCCATCATCAAGTGCCGGTCGTGCAG*
*GC57_Rs: CGCTGATTCTGTCTGTGTCATG*
*GC57_RsL: CATGCAATTATTGTGAGCAATACACACGCGCTTCCGCTGATTCTGTCTGTGTCATG*
*GC57_R1L: CATGCAATTATTGTGAGCAATACACACGCGCTTCTCTGGCGCACGCCCGGCGATGT*
*GC57_F1L: CATGCAATTATTGTGAGCAATACACACGCGCTTCCGTTCGTTTCTGATGATTTTGCTGC*

**Sequence 3, 36% GC, 21614-27348 of lambda DNA.**

*GC36_F: GCTCATGCCCACACAAGTG*
*GC36_FT7: TAATACGACTCACTATAGGGTCTGCCTGCAAACAGTACCG*
*GC36_R: AATGTCTGTTATGAGCGAGGAG*
*GC36_RT7: TAATACGACTCACTATAGGGTCAGCCAAGTTAATCAGATC*
*GC36_Fs: CCGCAATTTATTTGGCGGCAAC*
*GC36_FsL: CATGCAATTATTGTGAGCAATACACACGCGCTTCCGAGAGCATCAATATGCAATG*
*GC36_Rs: CTTCACCAATAAATTCATTAGTTC*
*GC36_RsL: CATGCAATTATTGTGAGCAATACACACGCGCTTCCTGGCTACCTGCATCAGGC*
*GC36_R1L: CATGCAATTATTGTGAGCAATACACACGCGCTTCTGCATCTACTCGTCGCGAACCGC*
*GC36_F1L: CATGCAATTATTGTGAGCAATACACACGCGCTTCGAGCATATTGCGCCGCTTCAGGATG*

## Section S2. Effect of the change in refractive index on experimental measurement

We analyzed the change in refractive index and the effect of this change on extension measurement. The refractive index of the objective oil ($n_0$) is about 1.518 at 22 °C (Olympus). To avoid the errors in RNA extensions caused by the different $n_b$ of buffers in Fig. 1$B-D$, we measured $n_b$ at different $c_{salt}$ using a refractometer (PAL-RI, ATAGO Japan) at 22 °C and corrected the RNA extensions according to the coefficient of $n_0/n_b$. The coefficient of $n_0/n_b$ decreased by about 0.7% when $c_{salt}$ was increased from 50 mM to 1 M for both NaCl and KCl (see the table below).

**Table S1. The reflective index of the buffer ($n_b$) and the coefficient $n_0/n_b$ at different $c_{salt}$.**

| Salt | 50 mM NaCl | 1 M NaCl | 50 mM KCl | 1 M KCl |
|---|---|---|---|---|
| $n_b$ | 1.3335 | 1.3436 | 1.3336 | 1.3435 |
| $n_0/n_b$ | 1.1384 | 1.1339 | 1.1383 | 1.1299 |

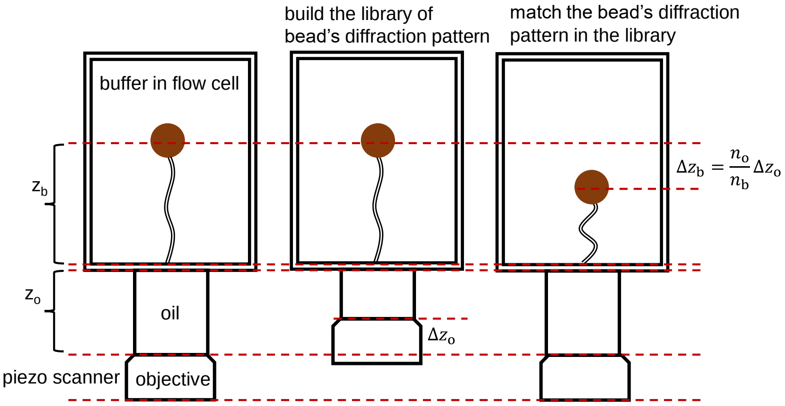


**Fig. S2.** Calculation of the RNA/DNA extensions based on the reflective indexes of the objective oil ($n_0$) and buffer in the flow cell ($n_b$).

As shown in Figure S2, before measuring twist-extension curves of RNA/DNA at each buffer condition, we built a library containing the diffraction pattern of each bead at each vertical position of objective using a piezo scanner which changes the thickness of objective oil by $\Delta z_o$. When measuring the twist-extension curves of RNA/DNA at each buffer condition, we determined the change in RNA/DNA extension ($\Delta z_b$) by matching the bead's the diffraction pattern in the library. As the same diffraction pattern of the bead meant the same optical length, we obtained $\Delta z_b = (n_0/n_b)\Delta z_0$.

The refractive index of buffer, $n_b$, also depends on temperature. As shown in a previous study (3), $n_b$ decreases $\sim 1.1 \times 10^{-4}$ per °C for 1 M KCl ($\sim$ 7% in mass concentration). In our temperature range from 22°C to 35°C, $\sim$ 7% decreased by $\sim$ 0.11%. Considering the $n_0$ of objective oil decreased $\sim 4 \times 10^{-4}$ per °C and decreased $\sim$ 0.52% in our temperature range, the coefficient of $n_0/n_b$ decreased by about $\sim$ 0.4%.

## Section S3. Experimental results of RNA and DNA twist changes induced by switching the ion type

To measure the RNA (or DNA) twist change induced by switching the ion type, we fixed the concentration at 150 mM (around physiological ionic strength) and measured the change in RNA (or DNA) twist change for the same RNA (or DNA) molecule when changing the ion type. The results are shown in Figure S3A. For DNA, the trend for DNA agrees with a previous study (4). Combining the result for RNA in Figure S3A. with the data in Fig. 2A, we obtained Figure S3B. For a given salt concentration, changing the ion type causes RNA twist change, which may be caused by different ion distribution patterns around RNA. See Sec. 10 for ion distribution patterns around RNA.

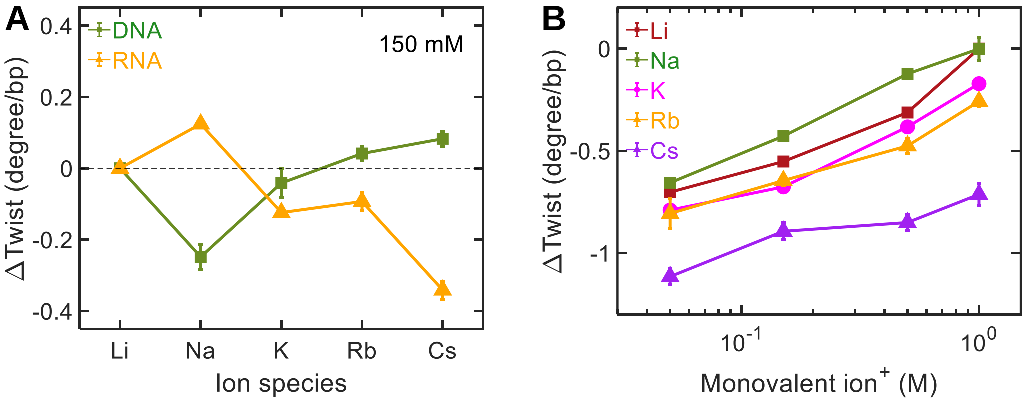


**Fig. S3.** (A) RNA and DNA twist changes with ion type and (B) RNA twist change as a function of salt concentration for different ion species from MT experiments. The error bars are standard deviations obtained from at least three measurements.

## Section S4. Experimental results of different RNA sequences

To examine the sequence effect, we carried out experiments for three different RNA sequences (as well as three same sequences for DNA except replacing U-base by T-base). The three sequences have CG contents of 36%, 43%, and 57%, respectively. Figure S4 shows noticeable changes in the twist-$c_{salt}$ curves for three RNA sequences. Such sequence effect may be caused by sequence dependences of nucleic acid mechanical properties. And we did not observe a clear trend with varying the CG percentage.

For temperature-induced RNA twist changes, we also carried out experiments using the above three sequences. Figure S5 shows that the sequence effect is quite weak for temperature-induced RNA twist changes at 1 M KCl. We did not observe a trend when varying the CG percentage.

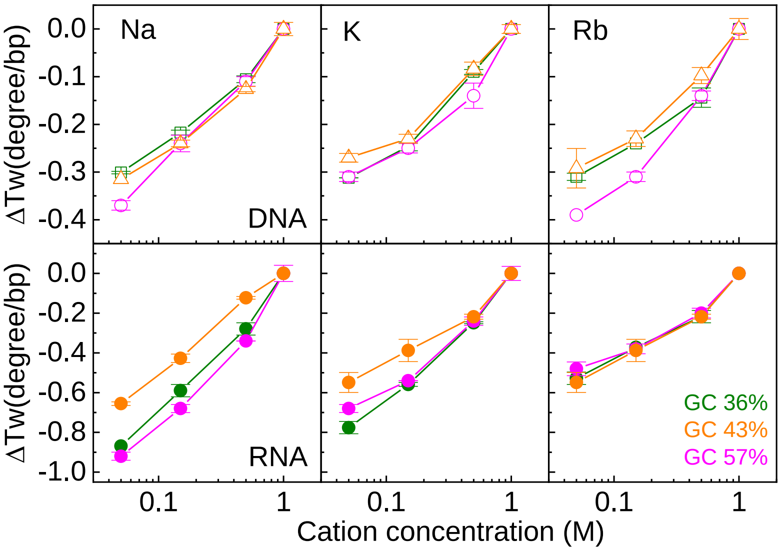


**Fig. S4.** RNA and DNA twist change as a function of the salt concentration for three salt species, $Na^+$, $K^+$, and $Rb^+$ from MT experiments. The error bars are standard deviations obtained from at least three measurements.

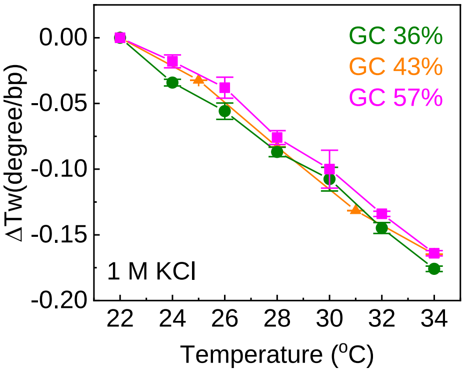


**Fig. S5.** RNA twist change as a function of the salt concentration for $K^+$ from MT experiments. The error bars are standard deviations obtained from at least three measurements.

## Section S5. Details of all-atom molecular dynamics simulations

The all-atom MD simulations were performed from the 25-bp A-form dsRNA with a sequence of CGACU CUACG GAAGG GCAUC UGCGC (5). The initial structure was built by the program 3DNA (6), and next placed in a rectangle box ($8 \times 8 \times 12.5$ nm) filled with TIP3P water molecules. Counterions of $Na^+$, $K^+$, $Rb^+$ described by the parameters from Joung and Cheatham model (7) were then added to neutralize the system. A certain number of salt ions were also added to produce the desired salt concentrations (0.05, 0.15, 0.3, 0.5 and 1 M). Each simulation system was started from energy minimization for 10000 steps, followed by 100 ps of thermalization in the isothermal ensemble and 10 ns of equilibration in the isothermic-isobaric ensemble. During the equilibration process, positional restraints were applied on dsRNA. Afterward, the system was treated for 600 ns MD simulation with restraints removed. The trajectory of last 500 ns simulation was used for data analysis.

All simulations were carried out with the GROMACS 2018.4 software (8) and OL3 force field (9). Periodic boundary conditions were applied in three dimensions. A 1.0 nm cut-off was used for van der Waals interactions and short-range electrostatic interactions. For long-range electrostatic interactions, Particle Mesh Ewald method was adopted (10). The LINCS algorithm (11) was also applied with a 2 fs time step using leap-frog integrator (12). The temperature of system was coupled through V-rescale thermostat (13) with a relaxation time $\tau = 0.1$ ps, and pressure was kept at 1 atm using Parrinello-Rahman pressure-coupling (14) with a relaxation time $\tau = 2.0$ ps and compressibility $4.5 \times 10^{-5}$ bar$^{-1}$. The trajectory was saved every 5000 steps, giving a total of 50000 conformations for analysis of each system.

## Section S6. Details of the calculation of $\Omega(\omega, G)$

Here we describe the calculation procedure of $\Omega(\omega, G)$ in Eq. 1. We collected $N_{\text{conf}} = 5 \times 10^4$ dsRNA conformations from a 500 ns trajectory of one MD simulation at a given salt condition. For each dsRNA conformation, we computed $\omega_i$ and $G_i$, where $i = 1, 2, \ldots, N_{\text{conf}}$ is the index of conformation. Then, these $N_{\text{conf}}$ data points of $\{\omega_i, G_i\}$ were grouped into $10 \times 10$ bins according to the values of $\omega$ and $G$. For example, one bin corresponds to $30.4^\circ < \omega \leq 33.7^\circ$ and $0.25$ nm $< G \leq 0.31$ nm. Finally, the number of data points in each bin is counted and recorded as $\Omega(\omega, G)$. When converting $\Omega(\omega, G)$ to $P_{\text{sim}}(\omega, G)$ using Eq. 1, we subtract a constant from $P_{\text{sim}}(\omega, G)$ to make its minimum value to be zero.

Table S2 displays the PMF obtained from the simulation with 1 M KCl. The data in Table S2 were used to determine $k_\omega^{\text{bp}}$, $k_G^{\text{bp}}$ and $k_{\omega G}^{\text{bp}}$ in Eq. 2 through fitting.

**Table S2. The two-dimensional potential of mean force (PMF) in 10×10 bins with respect to RNA twist angle and major groove width calculated from the 600 ns MD simulation at 1 M KCl. The unit of PMF is $k_{\text{B}}$T.**

| | 30.4°-30.7° | 30.7°-31.0° | 31.0°-31.3° | 31.3°-31.6° | 31.6°-31.9° | 31.9°-32.2° | 32.2°-32.5° | 32.5°-32.8° | 32.8°-33.1° | 33.1°-33.4° |
|---|---|---|---|---|---|---|---|---|---|---|
| 0.75-0.85 nm | 3.3114 | 2.6762 | 2.3062 | 2.2269 | 2.3165 | 2.6468 | 3.3694 | 4.3159 | | |
| 0.73-0.79 nm | 2.9891 | 2.0289 | 1.5290 | 1.3202 | 1.3495 | 1.7076 | 2.5306 | 3.2974 | 4.2782 | |
| 0.67-0.73 nm | 2.8736 | 1.9719 | 1.1905 | 0.8932 | 0.6445 | 0.8919 | 1.4760 | 2.2757 | 3.7239 | 4.6837 |
| 0.61-0.67 nm | 3.0632 | 2.0367 | 1.1639 | 0.5710 | 0.3051 | 0.3625 | 0.6885 | 1.4023 | 2.4150 | 4.0187 |
| 0.55-0.61 nm | 3.5851 | 2.2959 | 1.3357 | 0.5405 | 0.1571 | 0.0891 | 0.2313 | 0.7639 | 1.6632 | 2.9393 |
| 0.49-0.55 nm | 4.2067 | 3.0414 | 1.6156 | 0.7551 | 0.2120 | 0.0000 | 0.0571 | 0.4585 | 1.2232 | 2.2858 |
| 0.43-0.49 nm | 4.9350 | 3.4632 | 2.2034 | 1.1939 | 0.5093 | 0.1275 | 0.1211 | 0.3871 | 1.0579 | 2.0892 |
| 0.37-0.43 nm | 5.3768 | 3.8605 | 2.7783 | 1.7392 | 0.9920 | 0.5494 | 0.4368 | 0.6583 | 1.2267 | 2.0728 |
| 0.31-0.37 nm | 5.7823 | 5.6281 | 4.0775 | 2.7378 | 1.9393 | 1.3920 | 1.1905 | 1.2843 | 1.6339 | 2.2959 |
| 0.25-0.31 nm | | | 5.1761 | 4.2067 | 3.3399 | 2.4266 | 2.1491 | 2.0057 | 2.3811 | 2.7866 |

## Section S7. Dependences of the twist-groove coupling constants on the salt, sequence, and force field

Table S3 and Figure S6 present $k_\omega^{\mathrm{bp}}$, $k_G^{\mathrm{bp}}$, $k_{\omega G}^{\mathrm{bp}}$, $\omega_0$, and $G_0$ obtained by the fits to the simulation PMFs with different salt concentrations and species.

We also performed additional simulations using three different dsRNA constructs (15) or the Parmbsc0 force field (16). The calculated twist-groove coupling constants are shown in Table S4 and S7.

**Table S3. The twist-groove coupling per bp at different salt concentrations from 600 ns MD simulations. The uncertainties correspond to 95% confidence interval during the two-dimensional fitting.**

| NaCl (M) | $k_\omega^{bp}$ ($k_{\mathrm{B}}T/\deg^2$) | $k_G^{bp}$ ($k_{\mathrm{B}}T/\mathrm{nm}^2$) | $k_{\omega G}^{bp}$ ($k_BT/\deg\cdot\mathrm{nm}$) | $\omega_0$ (degree) | $G_0$ (nm) |
|---|---|---|---|---|---|
| 0.05 | 0.16±0.01 | 3.50±0.30 | 0.35±0.06 | 31.15 | 0.83 |
| 0.15 | 0.16±0.02 | 3.22±0.29 | 0.44±0.06 | 31.45 | 0.70 |
| 0.3 | 0.16±0.01 | 3.28±0.28 | 0.44±0.05 | 31.65 | 0.67 |
| 0.5 | 0.17±0.01 | 3.11±0.31 | 0.41±0.06 | 31.75 | 0.62 |
| 1 | 0.17±0.01 | 2.06±0.22 | 0.30±0.04 | 32.05 | 0.58 |
| KCl (M) | $k_\omega^{bp}$ ($k_BT/\deg^2$) | $k_G^{bp}$ ($k_BT/\mathrm{nm}^2$) | $k_{\omega G}^{bp}$ ($k_BT/\deg\cdot\mathrm{nm}$) | $\omega_0$ (degree) | $G_0$ (nm) |
| 0.05 | 0.14±0.01 | 3.89±0.29 | 0.41±0.05 | 31.55 | 0.70 |
| 0.15 | 0.17±0.01 | 3.78±0.25 | 0.46±0.04 | 31.65 | 0.65 |
| 0.3 | 0.17±0.01 | 3.61±0.27 | 0.43±0.05 | 31.85 | 0.6 |
| 0.5 | 0.17±0.01 | 3.56±0.25 | 0.41±0.05 | 31.95 | 0.58 |
| 1 | 0.18±0.01 | 3.61±0.28 | 0.43±0.05 | 32.15 | 0.52 |
| RbCl (M) | $k_\omega^{bp}$ ($k_BT/\deg^2$) | $k_G^{bp}$ ($k_BT/\mathrm{nm}^2$) | $k_{\omega G}^{bp}$ ($k_BT/\deg\cdot\mathrm{nm}$) | $\omega_0$ (degree) | $G_0$ (nm) |
| 0.05 | 0.17±0.01 | 3.94±0.28 | 0.50±0.05 | 31.75 | 0.67 |
| 0.15 | 0.17±0.01 | 3.72±0.18 | 0.42±0.03 | 31.95 | 0.62 |
| 0.3 | 0.18±0.01 | 3.50±0.20 | 0.39±0.04 | 32.05 | 0.57 |
| 0.5 | 0.17±0.01 | 3.44±0.19 | 0.39±0.03 | 32.15 | 0.55 |
| 1 | 0.19±0.01 | 3.39±0.13 | 0.34±0.02 | 32.25 | 0.5 |

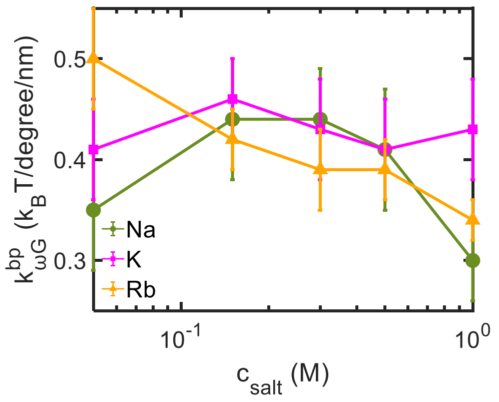


**Fig. S6.** The twist-groove coupling constant as a function of the concentration of NaCl, KCl or RbCl from simulations. The error bars are uncertainties corresponding to 95% confidence interval during the fitting.

**Table S4. The twist-groove coupling per bp for different sequences or force field obtained from 600 ns MD simulations at 1 M KCl. The uncertainties correspond to 95% confidence interval during the two-dimensional fitting.**

| Sequence | Force field | $k_\omega^{bp}$ $(k_BT/\deg^2)$ | $k_G^{bp}$ $(k_BT/\mathrm{nm}^2)$ | $k_{\omega G}^{bp}$ $(k_BT/\deg\cdot\mathrm{nm})$ |
|---|---|---|---|---|
| GGGGG-GGGGG-GGGGG-GGGGG-GGGGG | OL3 | 0.26±0.02 | 3.61±0.32 | 0.50±0.07 |
| GGGCG-GGCGG-GCGGG-CGGGC-GGGGG | OL3 | 0.16±0.01 | 3.11±0.17 | 0.34±0.04 |
| GGGCG-CGCGC-GCGCG-CGCGC-GCGGG | OL3 | 0.13±0.02 | 4.06±0.65 | 0.51±0.10 |
| GCAUC-UGGGC-UAUAA-AAGGG-CGUCG | OL3 | 0.18±0.01 | 4.72±0.27 | 0.33±0.05 |
| GAGAU-GCUAA-CCCUG-AUCGC-UGGAC | OL3 | 0.18±0.01 | 4.00±0.16 | 0.40±0.03 |
| GGGGG-CACGG-GGGGC-CUCGG-GAGGC | OL3 | 0.23±0.02 | 3.72±0.32 | 0.37±0.07 |
| CGACU-CUACG-GAAGG-GCAUC-UGCGC | ParmBsc0 | 0.17±0.01 | 4.33±0.26 | 0.37±0.04 |

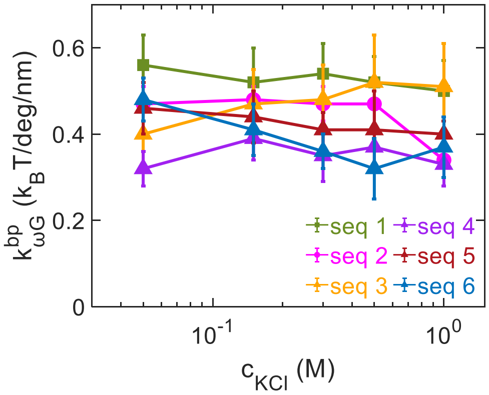


**Fig. S7.** The twist-groove coupling constant as a function of the concentration of KCl for different sequences from simulations. The error bars are uncertainties corresponding to 95% confidence interval during the fitting.

 

## Section S8. The effective twist rigidity for dsRNA

We can calculate the effective twist rigidity for a dsRNA molecule from $k_\omega^{\mathrm{bp}}$, $k_G^{\mathrm{bp}}$ and $k_{\omega G}^{\mathrm{bp}}$. Eq. 2 can be reformed as:

$$\begin{aligned} P &= \frac{1}{2}k_\omega^{\mathrm{bp}}(\Delta\omega)^2 + \frac{1}{2}k_G^{\mathrm{bp}}(\Delta G)^2 + k_{\omega G}^{\mathrm{bp}}\Delta\omega\Delta G \\ &= \frac{1}{2}k_G^{\mathrm{bp}}\left(\Delta G - \frac{k_{\omega G}^{\mathrm{bp}}}{k_G^{\mathrm{bp}}}\Delta\omega\right)^2 - \frac{1}{2}\frac{\left(k_{\omega G}^{\mathrm{bp}}\right)^2}{k_G^{\mathrm{bp}}}(\Delta\omega)^2 + \frac{1}{2}k_\omega^{\mathrm{bp}}(\Delta\omega)^2 \\ &= \frac{1}{2}k_G^{\mathrm{bp}}\left(\Delta G - \frac{k_{\omega G}^{\mathrm{bp}}}{k_G^{\mathrm{bp}}}\Delta\omega\right)^2 + \frac{1}{2}\left[k_\omega^{\mathrm{bp}} - \frac{\left(k_{\omega G}^{\mathrm{bp}}\right)^2}{k_G^{\mathrm{bp}}}\right]^2(\Delta\omega)^2. \end{aligned} \qquad \text{[S1]}$$

For every twist angle $\Delta\omega$, the major groove of dsRNA will be relaxed toward $\Delta G = \frac{k_{\omega G}^{\mathrm{bp}}}{k_G^{\mathrm{bp}}}\Delta\omega$ to minimize the first term, and then the energy cost is the second term $\frac{1}{2}\left[k_\omega^{\mathrm{bp}} - \frac{\left(k_{\omega G}^{\mathrm{bp}}\right.}{k_G^{\mathrm{bp}}}\right]^2\Bigg]^2(\Delta\omega)^2$. So, the effective twist rigidity is

$$\tilde{k}_\omega^{\mathrm{bp}} = k_\omega^{\mathrm{bp}} - \frac{\left(k_{\omega G}^{\mathrm{bp}}\right)^2}{k_G^{\mathrm{bp}}} \approx 0.129\ k_{\mathrm{B}}T/\mathrm{deg}^2. \qquad \text{[S2]}$$

While we define the twist rigidity for a base pair, another definition is $P = \frac{1}{2}\frac{C}{L}(\Delta\omega)^2$, where $P$ is the energy cost, $C$ is the twist rigidity, and $L$ is the DNA length. Then, $C = \tilde{k}_\omega^{\mathrm{bp}} \times L_{bp}$, where $L_{bp} \approx 0.338$ nm. After using the unit conversion $1\mathrm{rad} = 180/\pi\,\mathrm{deg}$ and $1\ k_{\mathrm{B}T} = 4.114\ \mathrm{pN}\cdot\mathrm{nm}$, eventually, we have $C = 0.129 \times \left(\frac{180}{\pi}\right)^2 \times 4.114 \times 0.338 = 589\ \mathrm{pN}\cdot\mathrm{nm}^2$. This value agrees with previous experimental result of $409\ \mathrm{pN}\cdot\mathrm{nm}^2$ (17) and simulation result of $310\ \mathrm{pN}\cdot\mathrm{nm}^2$ (18).

## Section S9. Theoretical calculation of $\Delta\omega$ using other values of P-P distance

We have used the P-P distance of $r = 0.6$ nm in the calculation of effective force in Eq. 7. Here, we show that other values of $r$ combining adjusting the rescale factor also produce agreeable results, as shown in Figure S8.

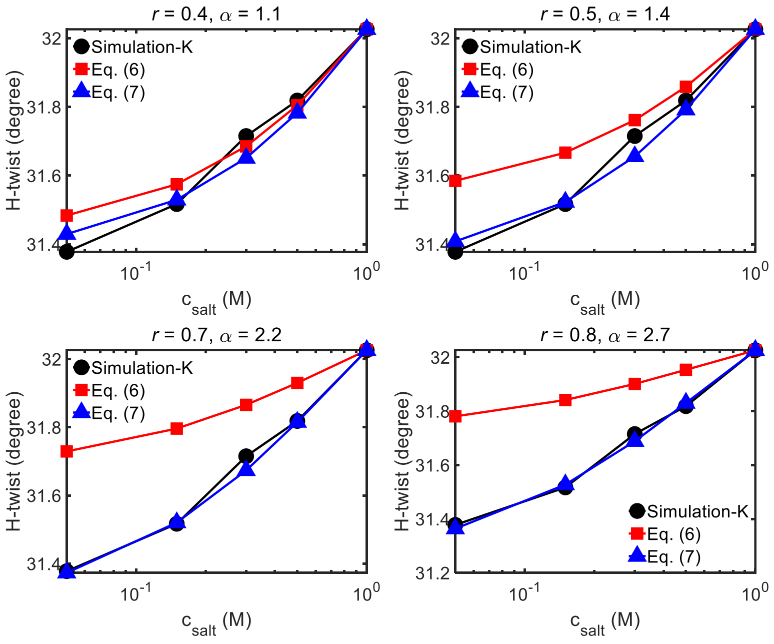


**Fig. S8.** Comparison of dsRNA twist changes from simulations of KCl and from Eq. 7 with different combinations of P-P distance, $r$ and rescale factor, $\alpha$.

## Section S10. Effect of ion type on the ion distribution around RNA and RNA twist change

As shown in Fig. 2, RNA twist change depends on not only the ion concentration but also the ion type. To understand the ion-type dependence, we analyzed the ion distributions around RNA for different ion types, as shown in Figures S9-S13.

To obtain an additional reason for the different deformation pathways between DNA and RNA, we compare ion distributions between DNA and RNA in Figure S14. It is obvious that RNA can capture much more ions in the major groove. Accordingly, RNA major groove width is significantly affected by ions, which mediates RNA twist change.

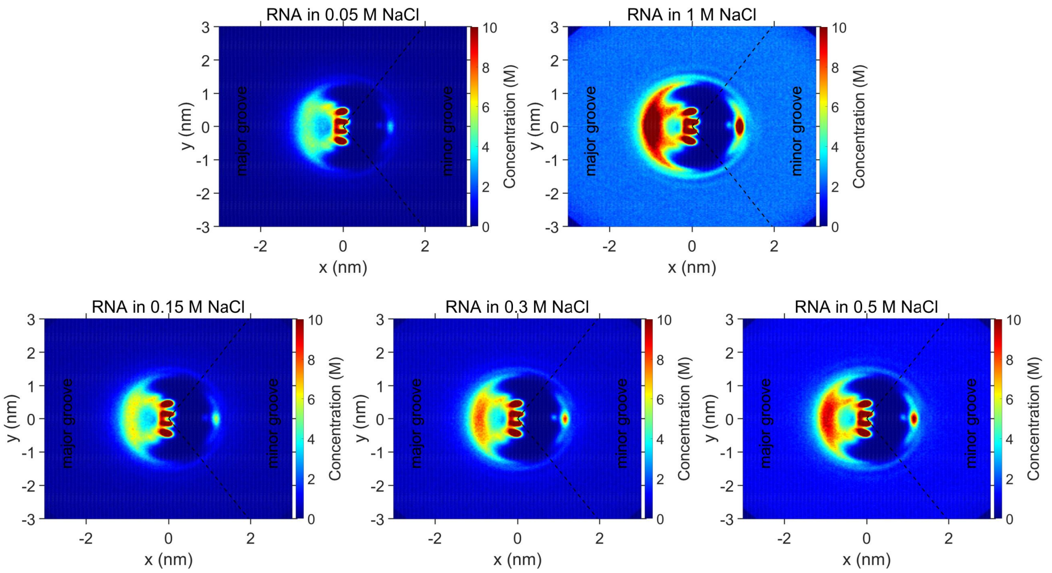


**Fig. S9.** Two-dimensional ion distribution of $Na^+$ around RNA from MD simulations.

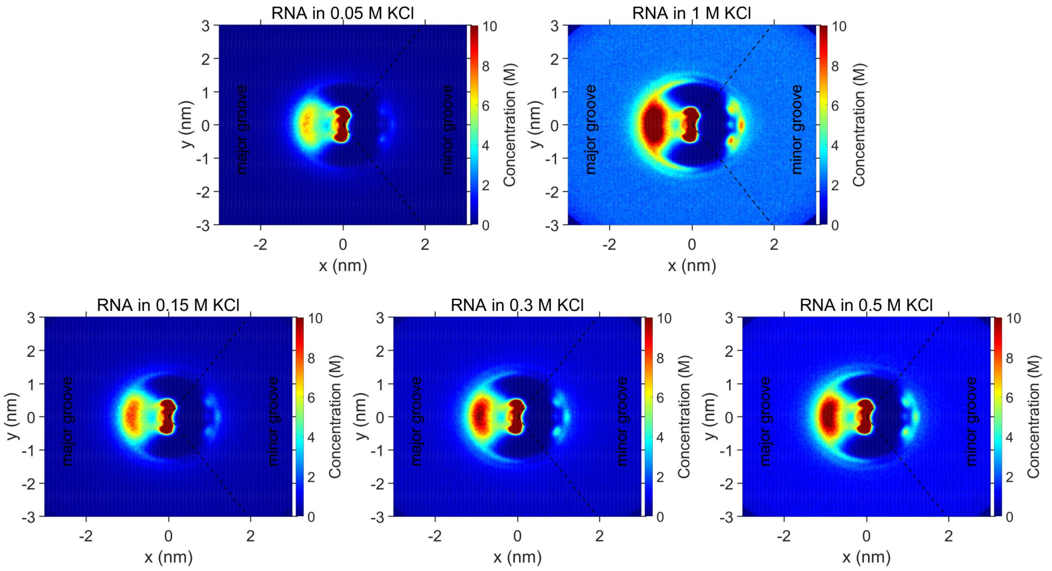


**Fig. S10.** Two-dimensional ion distribution of $K^+$ around RNA from MD simulations.

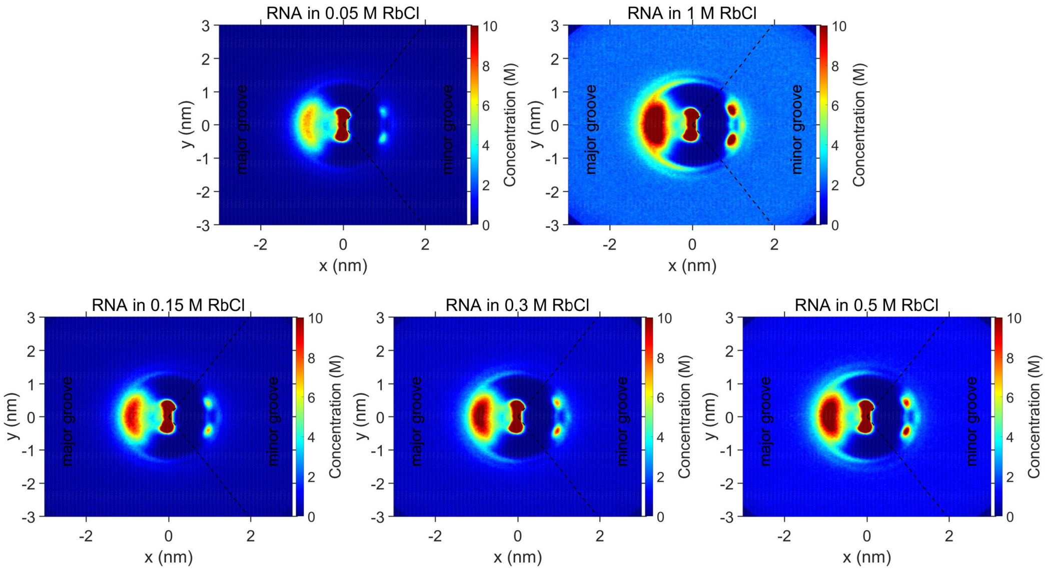


**Fig. S11.** Two-dimensional ion distribution of $\mathrm{Rb}^{+}$ around RNA from MD simulations.

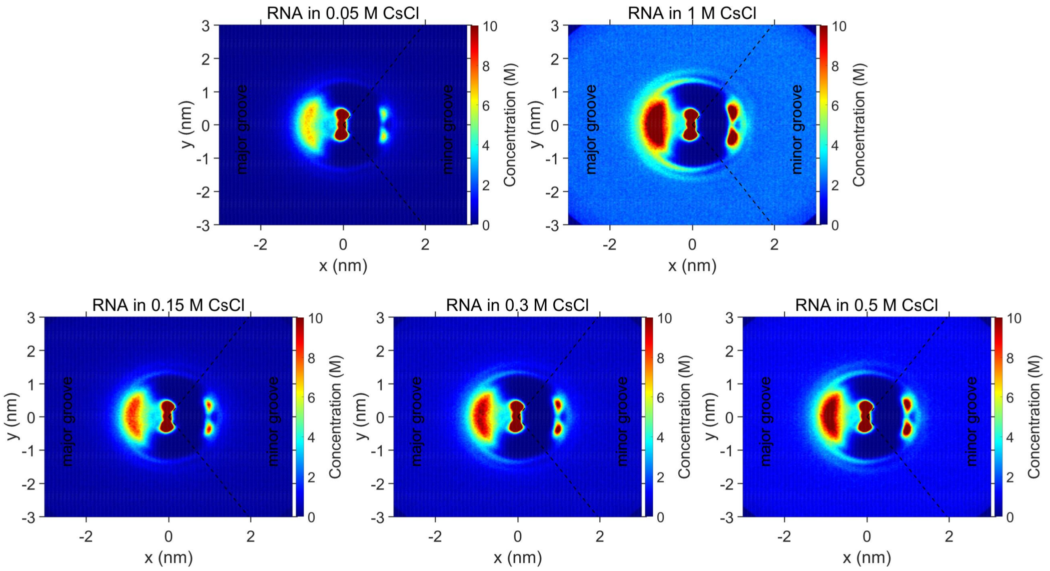


**Fig. S12.** Two-dimensional ion distribution of $\mathrm{Cs}^{+}$ around RNA from MD simulations.

**Table S5. Distribution of ions in phosphates, major groove, minor groove of RNA using the Joung-Cheatham model (7) and our modified Na+ parameters at varying salt concentrations from 600 ns MD simulations.**

| NaCl (M) | Phosphates | Major groove | Minor groove |
|---|---|---|---|
| 0.05 | 16.12% | 80.69% | 3.19% |
| 0.15 | 20.21% | 75.83% | 3.96% |
| 0.3 | 23.94% | 71.25% | 4.81% |
| 0.5 | 26.65% | 67.72% | 5.63% |
| 1.0 | 31.17% | 62.29% | 6.54% |
| KCl (M) | Phosphates | Major groove | Minor groove |
| 0.05 | 15.12% | 81.12% | 3.76% |
| 0.15 | 19.26% | 76.17% | 4.57% |
| 0.3 | 22.75% | 71.75% | 5.50% |
| 0.5 | 25.60% | 67.92% | 6.48% |
| 1.0 | 30.31% | 62.11% | 7.58% |
| RbCl (M) | Phosphates | Major groove | Minor groove |
| 0.05 | 14.60% | 79.94% | 5.46% |
| 0.15 | 18.49% | 75.16% | 6.35% |
| 0.3 | 21.74% | 70.92% | 7.34% |
| 0.5 | 24.72% | 66.82% | 8.46% |
| 1.0 | 29.32% | 61.59% | 9.09% |
| CsCl (M) | Phosphates | Major groove | Minor groove |
| 0.05 | 14.11% | 79.36% | 6.53% |
| 0.15 | 17.85% | 73.80% | 8.35% |
| 0.3 | 21.28% | 69.59% | 9.13% |
| 0.5 | 24.01% | 66.22% | 9.77% |
| 1.0 | 28.47% | 60.13% | 11.40% |

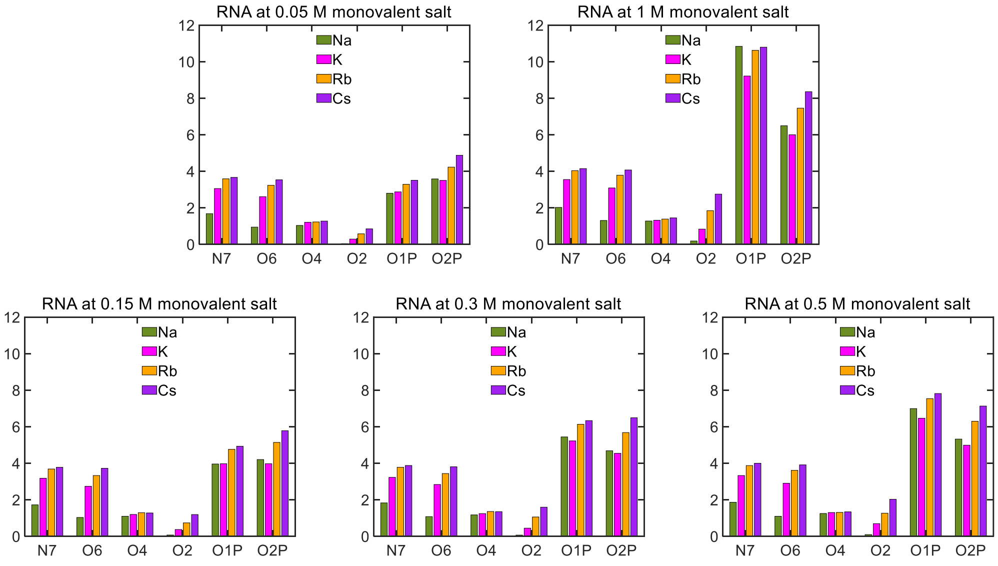


**Fig. S13.** Binding patterns between most frequent ion binding sites of RNA (N7 and O6 atoms of guanine, O4 atom of uracil, O2 atom of cytosine, O1P and O2P atoms of phosphate groups) (19) and monovalent ions ($\mathrm{Na}^+$, $\mathrm{K}^+$, $\mathrm{Rb}^+$, and $\mathrm{Cs}^+$) from MD simulations.

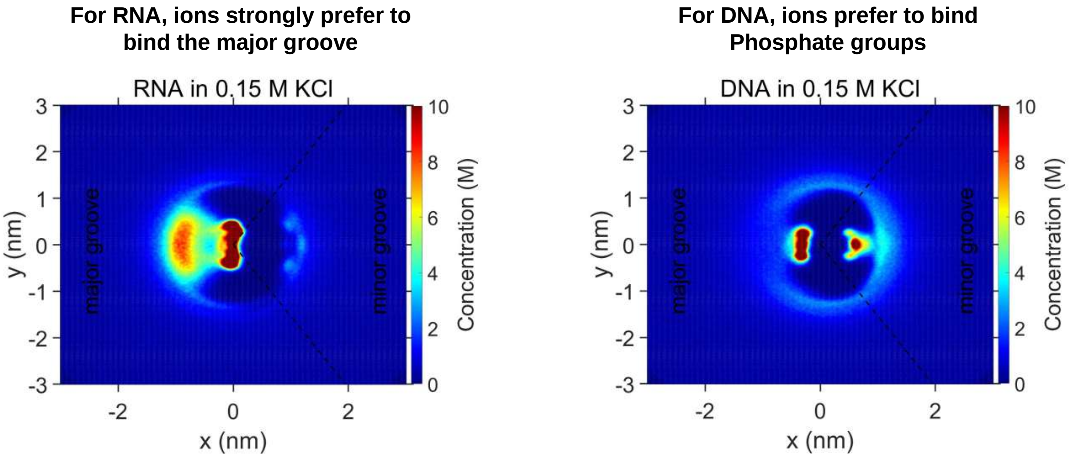


**Fig. S14.** Two-dimensional ion distribution of $\mathrm{K}^+$ around RNA and DNA from MD simulations at 0.15 M KCl.

## Section S11. Sequence effect on salt-induced RNA twist changes

We also carried out MD simulations for three different RNA sequences (Table S6, Y=pyrimidine, R=purine]) to examine the effects of base pair step number on RNA twist changes. As shown in Figure S15, higher pyrimidine-purine step content significantly increases RNA twist. The higher pyrimidine-purine step number also increases twist deformability, as proved by the smaller twist rigidity in Figure S16. This sequence-dependent result of RNA is consistent with the sequence dependence of DNA, where pyrimidine-purine basepair step has much higher twist flexibility than other basepair steps (20).

**Table S6. The twist-groove coupling per bp for different sequences or force field obtained from 600 ns MD simulations at 1 M KCl.**

| Number | Sequence | CG content | YR step content | RR step content | RY step content |
|---|---|---|---|---|---|
| Seq1 | $\mathrm{GGG}-(\mathrm{G})_{19}-\mathrm{GGG}$ | 100% | 0% | 100% | 0% |
| Seq2 | $\mathrm{GGG}-(\mathrm{CGGG})_{4}\mathrm{CGG}-\mathrm{GGG}$ | 100% | 28% | 50% | 22% |
| Seq3 | $\mathrm{GGG}-(\mathrm{CG})_{9}\mathrm{C}-\mathrm{GGG}$ | 100% | 50% | 0% | 50% |

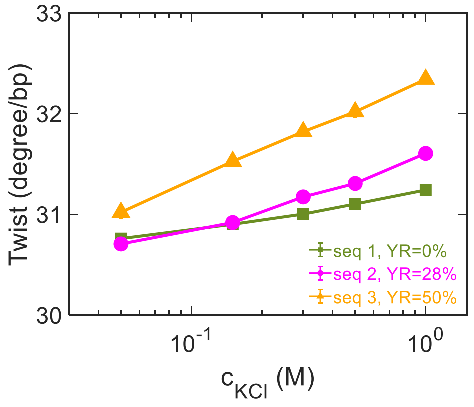


**Fig. S15.** RNA Twist as a function of the KCl concentration for sequences with different YR steps from MD simulations. The error bars denote the standard deviations obtained from the values of five equal intervals after equilibrium.

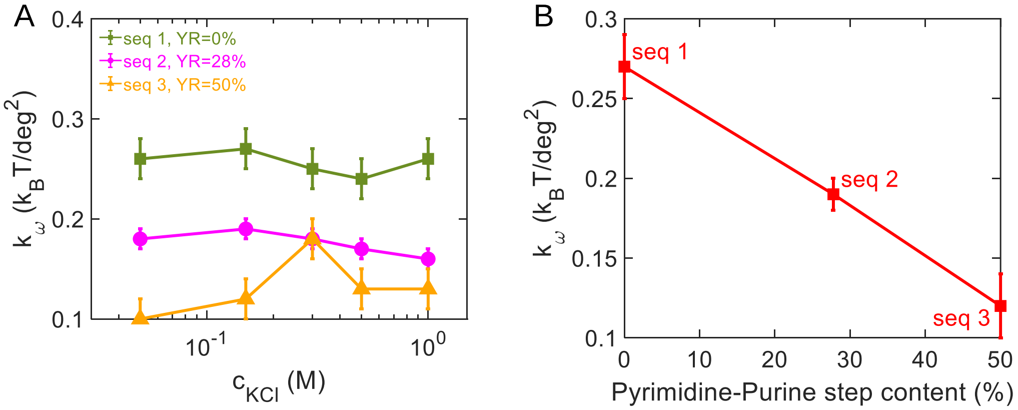


**Fig. S16.** RNA twist rigidity as a function of (A) KCl concentration for sequences with different YR steps and (B) YR step content at 0.15 M KCl from MD simulations. The error bars are uncertainties corresponding to 95% confidence interval during the fitting.

## Section S12. Discussion about other possible deformation pathways in dsRNA and dsDNA

Figure S17 illustrates the possible deformation pathways mediating salt-induced twist changes in dsDNA and dsRNA. The change in the salt concentration can affect the electrostatic repulsions between the phosphate groups across the diameter $D$, minor groove $G_{mi}$, and major groove $G_{ma}$. Then, the induced changes in $D$, $G_{mi}$, and $G_{ma}$ are possible to be transduced to the variation of twist $\omega$. The contribution of each deformation pathway to salt-induced twist change relies on both steps: (i) salt-induced change of $D$, $G_{mi}$, and $G_{ma}$, (ii) the coupling of $\omega$ with $D$, $G_{mi}$, and $G_{ma}$. To evaluate the relative weight of each deformation pathway, we carry out the following calculation to estimate (i) salt-induced change of $D$, $G_{mi}$, and $G_{ma}$, (ii) the coupling of $\omega$ with $D$, $G_{mi}$, and $G_{ma}$.

Salt-induced change of $D$, $G_{mi}$, and $G_{ma}$ should depend on the change in electrostatic force (just like $\Delta f_G$ in the main manuscript) and the stiffness of $D$, $G_{mi}$, and $G_{ma}$. Here, the stiffness of $D$, $G_{mi}$, and $G_{ma}$ can be quantified by the spring constant after assuming a harmonic energy cost of varying $D$, $G_{mi}$, and $G_{ma}$. The stiffness can be estimated through the fluctuation of $D$, $G_{mi}$, and $G_{ma}$ under thermal motion ($\sim k_{\mathrm{B}}T$)), as shown in Figure S18. Basically, a smaller fluctuation corresponds to a larger stiffness. For dsDNA, $G_{mi}$ and $G_{ma}$ are very stiff with small fluctuations, and $D$ has a moderate stiffness. For dsRNA, $G_{ma}$ is rather soft, and $G_{mi}$ and D are very stiff. To further demonstrate the softness of $G_{ma}$, we plot $G_{ma}$ as a function of the salt concentration in Figure S18.

The coupling of $\omega$ with $D$, $G_{mi}$, and $G_{ma}$ can be estimated through the correlation coefficients in Table S7. For dsDNA, $D$ is strongly correlated to $\omega$ with a correlation coefficient of -0.67; while $G_{mi}$ and $G_{ma}$ are moderately correlated to $\omega$ with correlation coefficients of -0.35 and -0.35, respectively. For dsRNA, $G_{ma}$ is strongly correlated to $\omega$ with a correlation coefficient of -0.47, and $G_{mi}$ is moderately correlated to $\omega$ with a correlation coefficient of 0.31. The correlation between $D$ and $\omega$ is also moderate.

Furthermore, we determined the $\omega - G_{ma}$ coupling constant for dsRNA and the $\omega - D$ coupling constant for dsDNA due to their important roles in salt-induced twist changes. The $\omega - G_{ma}$ coupling constant for dsRNA is $0.43 \pm 0.05$ $k_{\mathrm{B}}T/(\text{degree}\cdot\text{nm})$ per base pair, and the $\omega - D$ coupling constant for dsDNA is $4.5 \pm 0.8$ $k_{\mathrm{B}}T/(\text{degree}\cdot\text{nm})$per base pair. While the $\omega - G_{ma}$ coupling constant for dsRNA appears to be weaker than the $\omega - D$ coupling constant for dsDNA, dsRNA responds to the salt more profoundly than dsDNA. The reason is that dsRNA has a soft major groove width, relative to the stiff diameter of dsDNA. As illustrated in Figure S17, the combination of two steps determines the sensitivity of salt-induced twist change.

We also compare the magnitudes of the changes of many RNA structural parameters to verify the contribution of major groove width to twist changes when varying the ion concentration. To make the comparison reasonable, we normalize the change of a structural parameter by the standard deviation of the structural parameter under thermal fluctuations. As shown in Table S8, the major groove width has the greatest ratio compared to other three structural parameters. The changes of RNA diameter are so small (typically 0.002 nm) that they are not included in the table.

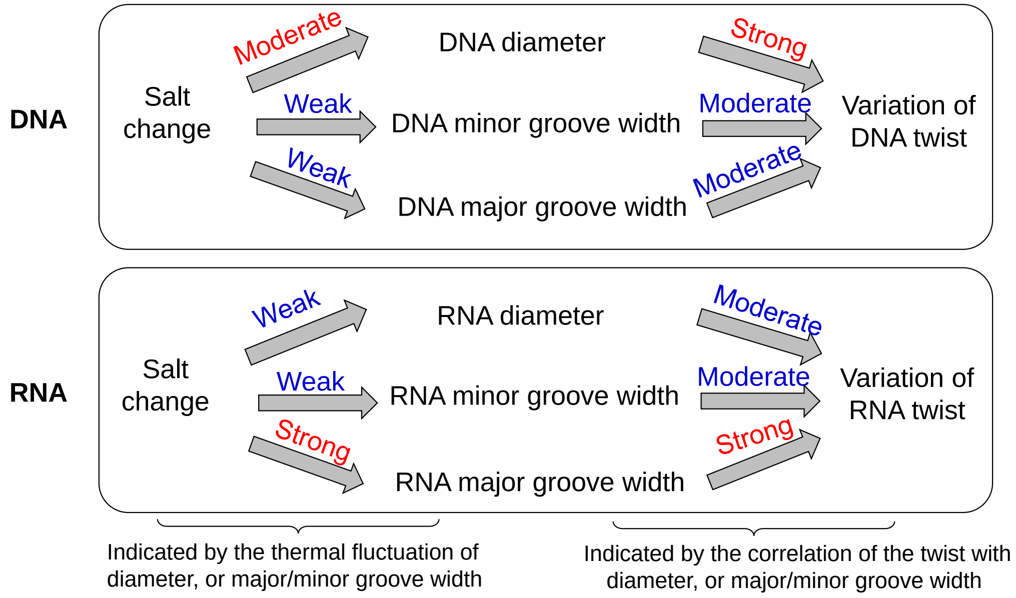


**Fig. S17.** Comparison of possible deformation pathways mediating salt-induced twist changes in dsDNA and dsRNA. The absolute values of correlation coefficients in the ranges of $[0\ \ 0.3)$, $[0.3\ \ 0.45)$, $[0.45\ \ 1]$ are considered as weak, moderate, and strong, respectively. The effects of the salt change on diameter and minor/major groove width are roughly classified into weak, moderate, and strong with no strict criteria.

**Table S7. Pearson correlation coefficients among twist, diameter, minor groove width and major groove width of dsDNA and dsRNA from our 600 ns MD simulation with 1 M KCl. Note that the data in the top-right triangle are identical to the data in the bottom-left triangle. The coefficients with the largest absolute values are marked in red.**

| DNA (1 M KCl) | Twist | Diameter | Minor groove width | Major groove width |
|---|---|---|---|---|
| Twist | 1.00 | -0.67 | -0.35 | -0.35 |
| Diameter | -0.67 | 1.00 | 0.10 | 0.42 |
| Minor groove width | -0.35 | 0.10 | 1.00 | -0.30 |
| Major groove width | -0.35 | 0.42 | -0.30 | 1.00 |
| RNA (1 M KCl) | Twist | Diameter | Minor groove width | Major groove width |
| Twist | 1.00 | -0.42 | 0.31 | -0.47 |
| Diameter | -0.42 | 1.00 | 0.03 | -0.05 |
| Minor groove width | 0.31 | 0.03 | 1.00 | -0.46 |
| Major groove width | -0.47 | -0.05 | -0.46 | 1.00 |

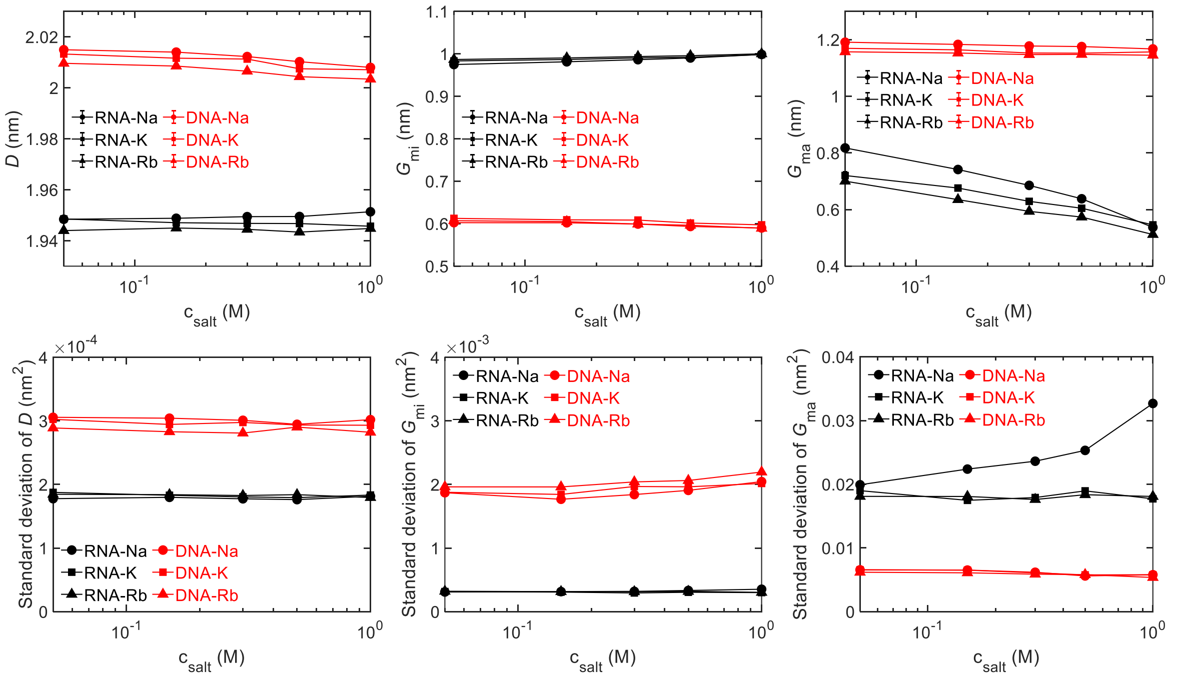


**Fig. S18.** Salt-induced change of $D$, $G_{mi}$, or $G_{ma}$ and their fluctuations as a function of salt concentration. Black and red solid lines represent simulation for dsRNA and dsDNA, respectively. Circle, square, and triangle symbols represent simulation from $\mathrm{Na}^+$, $\mathrm{K}^+$, and $\mathrm{Rb}^+$, respectively.

**Table S8. Normalized change of structural parameters by standard deviation among twist, major groove width, roll (bend), and tilt of dsRNA when varying salt concentrations from our 600 ns MD simulations.**

| **NaCl** | **Twist** | **Major groove width** | **Roll (bend)** | **Tilt** |
|---|---|---|---|---|
| Standard deviation at 0.15 M NaCl, $\delta$ | 0.4039 | 0.0224 | 1.7345 | 0.3503 |
| Change from 0.05 M to 0.15 M NaCl, $\Delta$ | 0.2249 | -0.0762 | 0.6667 | 0.0345 |
| **Ratio** $\Delta/\delta$ | **0.5567** | **-3.4074** | **0.3844** | **0.0984** |
| Standard deviation at 0.3 M NaCl, $\delta$ | 0.4096 | 0.0236 | 1.9993 | 0.3426 |
| Change from 0.05 M to 0.3 M NaCl, $\Delta$ | 0.4654 | -0.1323 | 1.3433 | 0.0034 |
| **Ratio** $\Delta/\delta$ | **1.1363** | **-5.6020** | **0.6719** | **0.0099** |
| Standard deviation at 0.5 M NaCl, $\delta$ | 0.4236 | 0.0253 | 2.0924 | 0.3576 |
| Change from 0.05 M to 0.5 M NaCl, $\Delta$ | 0.6551 | -0.1796 | 1.8242 | 0.0125 |
| **Ratio** $\Delta/\delta$ | **1.5464** | **-7.0961** | **0.8718** | **0.0350** |
| Standard deviation at 1 M NaCl, $\delta$ | 0.4056 | 0.0327 | 2.5852 | 0.3390 |
| Change from 0.05 M to 1 M NaCl, $\Delta$ | 0.9973 | -0.2805 | 2.8548 | -0.0023 |
| **Ratio** $\Delta/\delta$ | **2.4592** | **-8.5835** | **1.1043** | **-0.0068** |
| **KCl** | **Twist** | **Major groove width** | **Roll (bend)** | **Tilt** |
| Standard deviation at 0.15 M KCl, $\delta$ | 0.3997 | 0.0175 | 1.4434 | 0.3839 |
| Change from 0.05 M to 0.15 M KCl, $\Delta$ | 0.1391 | -0.0442 | 0.2303 | -0.0068 |
| **Ratio** $\Delta/\delta$ | **0.3480** | **-2.5321** | **0.1596** | **-0.0177** |
| Standard deviation at 0.3 M KCl, $\delta$ | 0.3662 | 0.0179 | 1.3653 | 0.3715 |
| Change from 0.05 M to 0.3 M KCl, $\Delta$ | 0.3372 | -0.0912 | 0.6744 | -0.0018 |
| **Ratio** $\Delta/\delta$ | **0.9208** | **-5.0935** | **0.4940** | **-0.0050** |
| Standard deviation at 0.5 M KCl, $\delta$ | 0.3699 | 0.0190 | 1.4111 | 0.3608 |
| Change from 0.05 M to 0.5 M KCl, $\Delta$ | 0.4407 | -0.1156 | 0.8827 | 0.0178 |
| **Ratio** $\Delta/\delta$ | **1.1913** | **-6.0960** | **0.6255** | **0.0492** |
| Standard deviation at 1 M KCl, $\delta$ | 0.3571 | 0.0177 | 1.4052 | 0.4133 |
| Change from 0.05 M to 1 M KCl, $\Delta$ | 0.6482 | -0.1743 | 1.4538 | 0.1408 |
| **Ratio** $\Delta/\delta$ | **1.8153** | **-9.8459** | **1.0346** | **0.3406** |
| **RbCl** | **Twist** | **Major groove width** | **Roll (bend)** | **Tilt** |
| Standard deviation at 0.15 M RbCl, $\delta$ | 0.3965 | 0.0181 | 1.4521 | 0.3388 |
| Change from 0.05 M to 0.15 M RbCl, $\Delta$ | 0.2327 | -0.0652 | 0.5749 | -0.1767 |
| **Ratio** $\Delta/\delta$ | **0.5868** | **-3.6053** | **0.3959** | **-0.5215** |
| Standard deviation at 0.3 M RbCl, $\delta$ | 0.3700 | 0.0176 | 1.4202 | 0.3487 |
| Change from 0.05 M to 0.3 M RbCl, $\Delta$ | 0.3755 | -0.1066 | 0.9287 | -0.1766 |
| **Ratio** $\Delta/\delta$ | **1.0148** | **-6.0581** | **0.6539** | **-0.5065** |
| Standard deviation at 0.5 M RbCl, $\delta$ | 0.3929 | 0.0184 | 1.4715 | 0.4686 |
| Change from 0.05 M to 0.5 M RbCl, $\Delta$ | 0.4744 | -0.1268 | 1.1751 | 0.0582 |
| **Ratio** $\Delta/\delta$ | **1.2074** | **-6.9069** | **0.7986** | **0.1242** |
| Standard deviation at 1 M RbCl, $\delta$ | 0.3338 | 0.0181 | 1.3828 | 0.3362 |
| Change from 0.05 M to 1 M RbCl, $\Delta$ | 0.6147 | -0.1883 | 1.6219 | -0.1649 |
| **Ratio** $\Delta/\delta$ | **1.8414** | **-10.4165** | **1.1730** | **-0.4905** |

## Section S13. RNA and DNA deformations induced by protein binding

Fig. 7A shows the results of dsRNA deformations within three dsRNA-protein complexes (21, 22). The reason why selected these three complexes is that in these three complexes, proteins mainly bind on the major grooves, which are likely to vary the major groove width. Basically, these three dsRNA-protein complexes are "clean" systems to analyze the correlation between the twist change and the major groove width during dsRNA deformations. In these three complexes, double-stranded RNA binding domains (dsRBD) mainly recognize the major groove by the N-terminal tip of helix $\alpha 2$ (23). For simplicity, we calculated the major groove width using the phosphate-phosphate distance perpendicular to the adjacent phosphate cubic spline curves across the major groove, which is similar to the groove parameters definition in 3DNA and Curves+ (6, 24). Overall, the results in Fig. 7A suggest that the protein-binding induced dsRNA deformations, in terms of $\Delta\omega$ and $\Delta G$, are along the direction of twist-groove coupling in dsRNA, which should reduce dsRNA deformation energies during protein binding. The dsRNA structural parameters for dsRNA-protein complexes were extracted directly from the structures in the PDB, while the standalone dsRNA structural parameters were obtained from our MD simulations of the dsRNA molecules with the specific sequences.

Then, we roughly estimate the contribution of the twist-groove coupling to the dsRNA deformation energy. The dsRNA deformation energy in the absence of the twist-groove coupling would be

$$P_{\text{nocouple}} \approx N_{bp}[\frac{1}{2}k_{\omega}^{\text{bp}}(\Delta\omega)^2 + \frac{1}{2}k_{G}^{\text{bp}}(\Delta G)^2]. \quad \text{[S3]}$$

Here, $N_{bp}$ is the number of RNA base pairs deformed by the protein binding. The dsRNA deformation energy with the twist-groove coupling is

$$P_{\text{couple}} \approx N_{bp}[\frac{1}{2}k_{\omega}^{\text{bp}}(\Delta\omega)^2 + \frac{1}{2}k_{G}^{\text{bp}}(\Delta G)^2 + k_{\omega G}^{\text{bp}}\Delta\omega\Delta G]. \quad \text{[S4]}$$

The contribution of the twist-groove coupling to the dsRNA deformation energy is

$$P_{\omega G} = P_{\text{couple}} - P_{\text{nocouple}} = N_{bp} \times k_{\omega G}^{\text{bp}}\Delta\omega\Delta G. \quad \text{[S5]}$$

**Table S9. DsRNA deformation energy reduced by twist-groove coupling obtained from 600 ns MD simulations**

| PDB ID | number of base pairs | $k_{\omega G}^{bp}$ ($k_{\text{B}}T/\deg\cdot\text{nm}$) | $\Delta\omega$(degree) | $\Delta G$(nm) | $P_{\omega G}(k_{\text{B}}T)$ |
|---|---|---|---|---|---|
| 2L2K | 6 | 0.44 | 1.97 | -0.14 | -0.746 |
| 2L3C | 10 | 0.44 | 6.43 | -0.06 | -1.705 |
| 3HTX | 14 | 0.44 | 0.91 | -0.07 | -0.404 |
| 2NUE | 16 | 0.44 | 1.79 | -0.13 | -1.655 |
| 3ADL | 4 | 0.44 | 2.17 | -0.19 | -0.732 |
| 6GPG | 7 | 0.44 | 1.57 | -0.34 | -1.629 |

We also analyze the normalized magnitudes of the changes of many structural parameters induced by protein binding. As shown by Table S11, the variations of major groove width appear to be most significant among other structural parameters.

Similarly, we selected three dsDNA-protein complexes to analyze dsDNA deformations upon protein binding. In these three complexes, proteins surround dsDNA molecules and affect dsDNA diameters. Fig. 7B shows the results for these three dsDNA-protein complexes (25, 26). The results suggest that the twist change and diameter change induced by protein binding are along the same direction as the twist-diameter coupling for DNA, which should reduce dsDNA deformation energies during protein binding. The contribution of the twist-diameter coupling to the dsDNA deformation energy is also estimated in Table S10.

**Table S10. DsDNA deformation energy reduced by twist-diameter coupling obtained from 600 ns MD simulations**

| PDB ID | number of base pairs | $k_{\omega D}^{bp}$ ($k_{\text{B}}T/\deg\cdot\text{nm}$) | $\Delta\omega$(degree) | $\Delta D$(nm) | $P_{\omega D}(k_{\text{B}}T)$ |
|---|---|---|---|---|---|
| 1BY4 | 8 | 3.85 | 0.34 | -0.024 | -0.248 |
| 7AIB | 23 | 3.85 | 1.41 | -0.067 | -8.397 |
| 7AIC | 23 | 3.85 | 1.53 | -0.074 | -9.977 |
| 1JJ4 | 16 | 3.85 | 0.51 | -0.059 | -1.155 |
| 2R5Z | 4 | 3.85 | 0.46 | -0.046 | -1.041 |
| 6XWH | 7 | 3.85 | 0.51 | -0.067 | -1.307 |

**Table S11. Normalized change of structural parameters by standard deviation among twist, major groove width, roll (bend), and tilt of dsRNA-protein complexes with protein binding from our 600 ns MD simulations.**

| PDB ID | | Twist | Major groove width | Roll (bend) | Tilt |
|---|---|---|---|---|---|
| 2L2K | Standard deviation before protein binding, $\delta$ | 1.3263 | 0.0533 | 4.4762 | 1.0349 |
| | Change after protein binding, $\Delta$ | 1.9717 | -0.1433 | -5.7962 | -0.6293 |
| | **Ratio** $\Delta/\delta$ | **1.4866** | **-2.6904** | **-1.2949** | **-0.6081** |
| 2L3C | Standard deviation before protein binding, $\delta$ | 2.7266 | 0.0190 | 4.6127 | 0.8855 |
| | Change after protein binding, $\Delta$ | 6.4260 | -0.0603 | -4.6380 | 0.7801 |
| | **Ratio** $\Delta/\delta$ | **2.3568** | **-3.1722** | **-1.0055** | **0.8809** |
| 2NUE | Standard deviation before protein binding, $\delta$ | 0.5264 | 0.0180 | 1.9388 | 0.4308 |
| | Change after protein binding, $\Delta$ | 1.7935 | -0.1311 | -1.1208 | 0.5806 |
| | **Ratio** $\Delta/\delta$ | **3.4068** | **-7.2730** | **-0.5781** | **1.3479** |
| 3ADL | Standard deviation before protein binding, $\delta$ | 3.5994 | 0.0819 | 9.7940 | 2.6113 |
| | Change after protein binding, $\Delta$ | 2.1716 | -0.1914 | 0.5919 | -0.2846 |
| | **Ratio** $\Delta/\delta$ | **0.6033** | **-2.3378** | **0.0604** | **-0.1090** |
| 3HTX | Standard deviation before protein binding, $\delta$ | 0.5184 | 0.0238 | 2.2595 | 0.4876 |
| | Change after protein binding, $\Delta$ | 0.9132 | -0.0719 | -2.6882 | 0.3547 |
| | **Ratio** $\Delta/\delta$ | **1.7615** | **-3.0238** | **-1.1897** | **0.7275** |
| 6GPG | Standard deviation before protein binding, $\delta$ | 1.7086 | 0.0566 | 5.3221 | 2.1035 |
| | Change after protein binding, $\Delta$ | 1.5671 | -0.3376 | -1.4819 | -1.1037 |
| | **Ratio** $\Delta/\delta$ | **0.9172** | **-5.9613** | **-0.2784** | **-0.5247** |

## Section S14. Modification of the force field parameters for $Na^+$

As shown in Fig. 2, the simulation results of $K^+$ and $Rb^+$ agree well with the experimental results, while the simulation results of $Na^+$ deviate from the experimental results. Considering that all-atom MD force fields keep evolving to better match experiments, we take advantage of our experimental results and modify the force field parameters for better agreement with experiments. We hope such attempts of modification can be useful in future development of force field parameters for $Na^+$.

The force field parameters of $Na^+$ include the sigma and epsilon for the Lennard-Jones interaction. We modified the sigma and epsilon independently, carried out new MD simulations, and then compared simulation and experimental results (Figure S19 and Table S12). It is interesting that increasing sigma can substantially increase the number of $Na^+$ in major groove width (Table S13), which is probably the reason why the modified LJ parameter can better match experimental results. Eventually, we find that slightly increasing the sigma from 0.243928 nm to 0.29000 nm (modified parameters 4) can achieve a good agreement.

**Table S12. Comparison of original parameters using the Joung-Cheatham model (7) and our modified parameters obtained from 600 ns MD simulations.**

| $Na^+$ | Sigma (nm) | Epsilon (kJ/mol) |
|---|---|---|
| Joung-Cheatham model [5] | 0.243928 | 0.365846 |
| Modified parameters 1 | 0.243928 | 0.180000 |
| Modified parameters 2 | 0.243928 | 0.540000 |
| Modified parameters 3 | 0.270000 | 0.365846 |
| Modified parameters 4 | 0.290000 | 0.365846 |

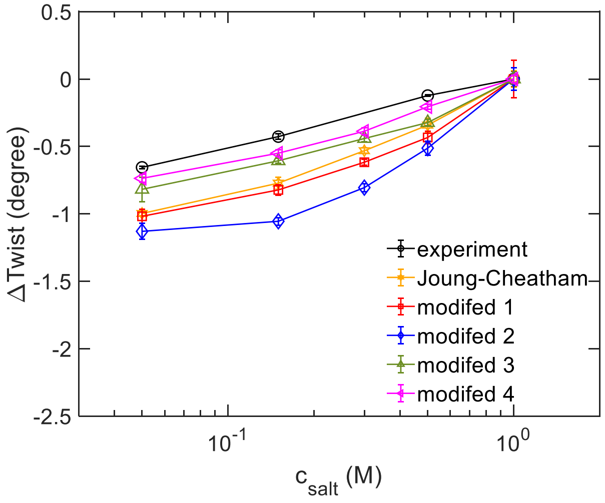


**Fig. S19.** Comparison of experimental and simulation twist change as a function of NaCl salt concentration using our modified $Na^+$ parameters. The error bars denote the standard deviations obtained from the values of five equal intervals after equilibrium.

**Table S13. Distribution of ions in phosphates, major groove, minor groove of RNA using the Joung-Cheatham model (7) and our modified Na+ parameters at varying salt concentrations from 600 ns MD simulations.**

| 0.05 M NaCl | Sigma (nm) | Epsilon (kJ/mol) | Phosphates | Major groove | Minor groove |
|---|---|---|---|---|---|
| Joung-Cheatham model | 0.243928 | 0.365846 | 3.57 | 17.86 | 0.71 |
| Modified 1 | 0.243928 | 0.180000 | 3.91 | 19.15 | 0.76 |
| Modified 2 | 0.243928 | 0.540000 | 4.16 | 18.77 | 1.16 |
| Modified 3 | 0.270000 | 0.365846 | 3.74 | 18.90 | 1.16 |
| Modified 4 | 0.290000 | 0.365846 | 3.72 | 19.40 | 0.64 |
| 0.15 M NaCl | Sigma (nm) | Epsilon (kJ/mol) | Phosphates | Major groove | Minor groove |
| Joung-Cheatham model | 0.243928 | 0.365846 | 5.29 | 19.82 | 1.03 |
| Modified 1 | 0.243928 | 0.180000 | 5.82 | 21.30 | 1.13 |
| Modified 2 | 0.243928 | 0.540000 | 5.88 | 21.73 | 1.27 |
| Modified 3 | 0.270000 | 0.365846 | 5.70 | 21.28 | 1.13 |
| Modified 4 | 0.290000 | 0.365846 | 5.50 | 21.21 | 1.08 |
| 0.3 M NaCl | Sigma (nm) | Epsilon (kJ/mol) | Phosphates | Major groove | Minor groove |
| Joung-Cheatham model | 0.243928 | 0.365846 | 7.25 | 21.59 | 1.46 |
| Modified 1 | 0.243928 | 0.180000 | 7.81 | 23.18 | 1.51 |
| Modified 2 | 0.243928 | 0.540000 | 7.85 | 23.71 | 1.55 |
| Modified 3 | 0.270000 | 0.365846 | 7.63 | 23.04 | 1.48 |
| Modified 4 | 0.290000 | 0.365846 | 7.39 | 22.86 | 1.40 |
| 0.5 M NaCl | Sigma (nm) | Epsilon (kJ/mol) | Phosphates | Major groove | Minor groove |
| Joung-Cheatham model | 0.243928 | 0.365846 | 9.07 | 23.06 | 1.92 |
| Modified 1 | 0.243928 | 0.180000 | 9.87 | 24.94 | 2.14 |
| Modified 2 | 0.243928 | 0.540000 | 10.08 | 25.72 | 2.15 |
| Modified 3 | 0.270000 | 0.365846 | 9.80 | 24.81 | 1.93 |
| Modified 4 | 0.290000 | 0.365846 | 9.62 | 24.70 | 1.88 |
| 1 M NaCl | Sigma (nm) | Epsilon (kJ/mol) | Phosphates | Major groove | Minor groove |
| Joung-Cheatham model | 0.243928 | 0.365846 | 13.33 | 26.64 | 2.80 |
| Modified 1 | 0.243928 | 0.180000 | 14.11 | 28.89 | 3.35 |
| Modified 2 | 0.243928 | 0.540000 | 14.28 | 29.97 | 3.17 |
| Modified 3 | 0.270000 | 0.365846 | 14.41 | 28.55 | 2.88 |
| Modified 4 | 0.290000 | 0.365846 | 14.07 | 28.36 | 2.59 |

## Section S15. The role of twist-groove coupling in temperature-induced twist change

We performed MD simulations to analyze the effects of temperature on RNA twist. Similar to the temperature-induced DNA twist change, our MD simulations for temperature-induced RNA twist change yield PMF with respect to the major groove width under various temperatures (Figure S20), which allows us to separate the contributions of interaction energy $U$ and conformational entropy $S$ to RNA free energy:

$$F(G) = U(G) - TS(G), \quad \text{[S6]}$$

where $U(G)$ is the internal energy and $S(G)$ is the entropy, both as a function of the major groove width. Taking the PMF in 1 M KCl at 22 °C as a reference, we subtract it from the PMF at one another temperature (RNA in 1 M KCl at 27 °C) to give the PMF difference.

$$F(27\ ^\circ\mathrm{C}) - F(22\ ^\circ\mathrm{C}) = -5S(G) + a, \quad \text{[S7]}$$

where $a$ is the constant independent of $G$. Dividing Eq. S7 by the prefactor of $S(G)$, we obtain $S(G)$ plus a constant:

$$[F(22\ ^\circ\mathrm{C}) - F(27\ ^\circ\mathrm{C})]/5 = S(G) - a/5. \quad \text{[S8]}$$

Here we just need how $S$ changes as a function of $G$ instead of the absolute value of $S$. As shown in Fig. 5B, we find that the extracted RNA conformational entropy $S(G)$ indeed increases with the major groove width $G$ linearly, specifically in the range of $G \in [0.4, 0.8]$ nm. We then performed the following calculations to connect the major groove width-dependent entropy $S(G)$ with temperature-dependent twist change $\omega(T)$. The change in temperature, $\Delta T$, exerts an effective force on RNA major groove width, $\Delta f_T(\Delta T)$:

$$\begin{aligned} \Delta f_T &= -\frac{\partial \Delta F}{\partial G} = \frac{\partial (S\Delta T)}{\partial G} = \Delta T\frac{\partial S}{\partial G} \approx k_{SG} \times \Delta T \\ with\ k_{SG} &\equiv \frac{\partial S}{\partial G} \approx 0.024\ \mathrm{kJ/(mol \cdot {}^\circ C \cdot nm)}. \end{aligned} \quad \text{[S9]}$$

The value of $\partial S/\partial G \approx 0.024\ \mathrm{kJ/(mol \cdot K \cdot nm)}$ was obtained from the simulation result in Figure S20. In the main manuscript, we have obtained the relationship between the effective force that tends to change the major groove width and hence twist. Accordingly, we have:

$$\Delta\omega_{\mathrm{bp}} = \frac{-k_{\omega G}^{\mathrm{bp}}}{k_{\omega}^{\mathrm{bp}} k_{G}^{\mathrm{bp}} - (k_{\omega G}^{\mathrm{bp}})^2}\Delta f_T = \frac{-k_{\omega G}^{\mathrm{bp}}}{k_{\omega}^{\mathrm{bp}} k_{G}^{\mathrm{bp}} - (k_{\omega G}^{\mathrm{bp}})^2} k_{SG}\Delta T. \quad \text{[S10]}$$

It means the temperature-dependent RNA twist change has a coefficient $k_T$:

$$k_T^{\mathrm{bp}} \equiv \frac{\Delta\omega_{\mathrm{bp}}}{\Delta T} = \frac{-k_{\omega G}^{\mathrm{bp}}}{k_{\omega}^{\mathrm{bp}} k_{G}^{\mathrm{bp}} - (k_{\omega G}^{\mathrm{bp}})^2} k_{SG}. \quad \text{[S11]}$$

At $T = 22$ °C, using the relationship 1 $k_{\mathrm{B}}T \approx 2.454$ kJ/mol, we made the conversion: $k_{SG} \approx 0.024\ \mathrm{kJ/(mol \cdot {}^\circ C \cdot nm)} \approx 0.010\ k_{\mathrm{B}}T/(^\circ\mathrm{C} \cdot \mathrm{nm})$. With $\frac{-k_{\omega G}^{\mathrm{bp}}}{k_{\omega}^{\mathrm{bp}} k_{G}^{\mathrm{bp}} - (k_{\omega G}^{\mathrm{bp}})^2} \approx -1.153\ \mathrm{deg \cdot nm}/k_{\mathrm{B}}T$. Eventually, we predict

$$k_T^{\mathrm{bp}} \approx -1.153 \times 0.010\ \mathrm{deg/^\circ C} \approx -0.012\ ^\circ/(^\circ\mathrm{C} \cdot \mathrm{bp}). \quad \text{[S12]}$$

The above coefficient agrees with our experimental result of $-0.014\ ^\circ/(^\circ\mathrm{C} \cdot \mathrm{bp})$ in 1 M KCl, as shown in Fig. 5A.

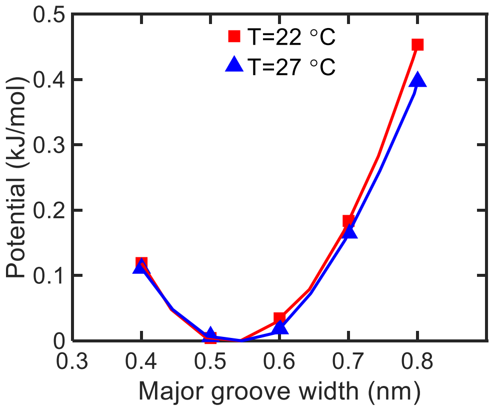


**Fig. S20.** The PMFs of the RNA major groove width from simulation results at 22 °C and 27 °C, respectively.

## Section S16. Comparison of temperature-induced RNA and DNA twist changes

In Figure S21, we compare temperature dependence for DNA and RNA. The slopes are similar for DNA and RNA. The mechanisms are presented in the flowchart.

The reason why RNA and DNA have opposite twist-stretch coupling is illustrated by the flowchart (Figure S22). The opposition comes from the first step (stretching → major groove width and stretching → diameter). For RNA, both stretching and major groove width share the longitudinal direction, while for DNA, stretching and diameter correspond to longitudinal and transversal directions, respectively. Due to the volume conservation, DNA sizes in longitudinal and transversal directions are negatively correlated.

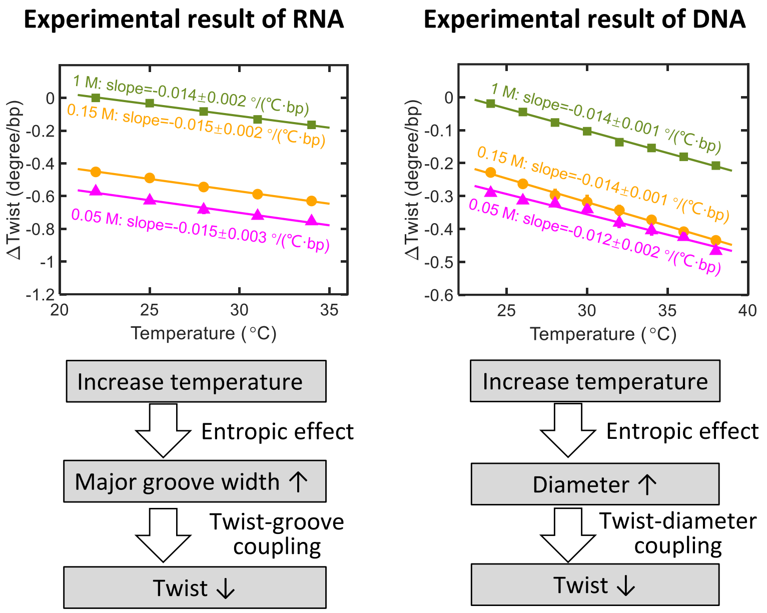


**Fig. S21.** Compare temperature dependence for RNA and DNA.

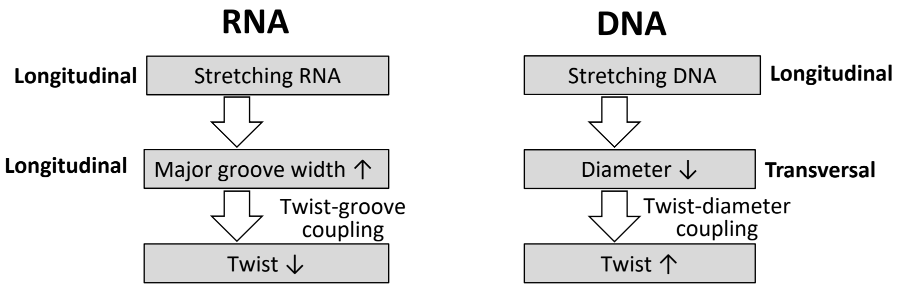


**Fig. S22.** Illustration of opposite twist-stretch couplings in RNA and DNA.

## Section S17. A simple helical model for RNA twist-groove coupling

RNA twist-groove coupling and DNA twist-diameter coupling can be simply and roughly explained using a simple helical model. Two adjacent bases are connected by a fragment of sugar-phosphate backbone, whose length, $s$, is roughly fixed. The length, $s$, can be written as $s = \sqrt{(D\omega/2)^2 + h^2}$, where $h$ is the helical rise per bp. Under the condition that $s$ is fixed, there are two cases. (i) When $h$ is fixed, $\omega$ and $D$ are negatively correlated, i.e., twist-diameter coupling in DNA. This explanation was used in our previous study (27). (ii) When $D$ is fixed, $\omega$ and $h$ are negatively correlated. Considering the strong positive correlation between $h$ and the major groove width $G$, one obtains the negative correlation between $\omega$ and $G$ in RNA (Fig. 3A, 3C).

## Section S18. Confirmation of RNA twist-groove coupling using MD simulations with external forces on major grooves

We performed additional simulations with artificial forces to enlarge RNA major grooves and then observe RNA twist changes. For each pair of P atoms across the RNA major groove, we imposed two springs that pull each P atom toward the other P atom (see the left panel in Figure S23). We set the spring constant to be negative such that the spring force tends to enlarge RNA major grooves. We set five values of spring constant so that the spring forces equal 1, 2, 3, 4, and 5 pN, respectively. The distance for the P-P pair across RNA major groove is around 0.8353 nm, and hence the spring constants are roughly -0.6022, -1.2044, -1.8066, -2.4088, and -3.011 $\mathrm{kJ} \cdot \mathrm{mol}^{-1} \cdot \mathrm{nm}^{-2}$. Figure S23 shows the corresponding RNA twist changes for these five springs. Each data point corresponds to a simulation of 600 ns. The scatter points are close to the ones for salt-induced RNA twist changes, which supports that the variation of major groove width plays a major role in mediating RNA twist change.

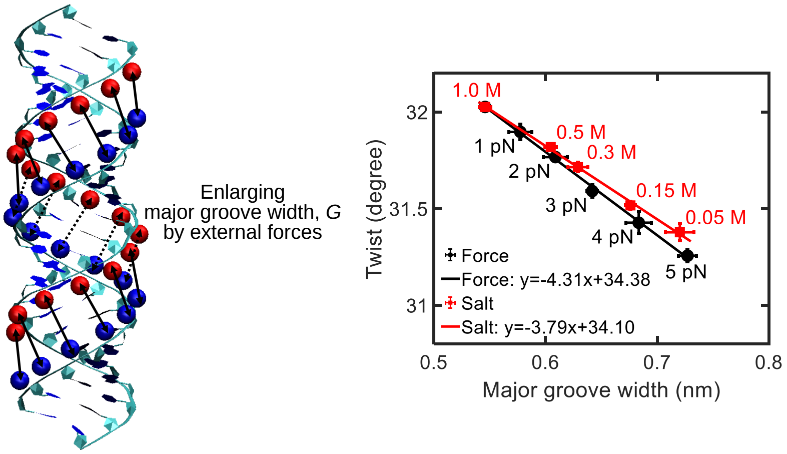


**Fig. S23.** Illustration of adding force to enlarge major groove of RNA (left) and the force-induced twist-groove curve agrees with that obtained from salt variation by 600 ns MD simulations (right).

## References


1. H Fu, et al., Opposite effects of high-valent cations on the elasticities of DNA and RNA duplexes revealed by magnetic tweezers. *Phys. Rev. Lett.* **124**, 058101 (2020).
2. XW Qiang, et al., Multivalent cations reverse the twist-stretch coupling of RNA. *Phys. Rev. Lett.* **128**, 108103 (2022).
3. CY Tan, YX Huang, Dependence of refractive index on concentration and temperature in electrolyte solution, polar solution, nonpolar solution, and protein solution. *J. Chem. Eng. Data* **60**, 2827–2833 (2015).
4. S Cruz-León, et al., Twisting DNA by salt. *Nucleic Acids Res.* **50**, 5726–5738 (2022).
5. RS Mathew-Fenn, R Das, PA Harbury, Remeasuring the double helix. *Science* **322**, 446–449 (2008).
6. XJ Lu, WK Olson, 3DNA: a software package for the analysis, rebuilding and visualization of three-dimensional nucleic acid structures. *Nucleic Acids Res.* **31**, 5108–5121 (2003).
7. IS Joung, TE Cheatham III, Determination of alkali and halide monovalent ion parameters for use in explicitly solvated biomolecular simulations. *J. Phys. Chem. B* **112**, 9020–9041 (2008).
8. MJ Abraham, et al., Gromacs: High performance molecular simulations through multi-level parallelism from laptops to supercomputers. *SoftwareX* **1**, 19–25 (2015).
9. M Zgarbová, et al., Refinement of the cornell et al. nucleic acids force field based on reference quantum chemical calculations of glycosidic torsion profiles. *J. Chem. Theory Comput.* **7**, 2886–2902 (2011).
10. U Essmann, et al., A smooth particle mesh ewald method. *J. Chem. Phys.* **103**, 8577–8593 (1995).
11. B Hess, H Bekker, HJ Berendsen, JG Fraaije, Lincs: a linear constraint solver for molecular simulations. *J. Comput. Chem.* **18**, 1463–1472 (1997).
12. RW Hockney, S Goel, J Eastwood, Quiet high-resolution computer models of a plasma. *J. Comput. Phys.* **14**, 148–158 (1974).
13. G Bussi, D Donadio, M Parrinello, Canonical sampling through velocity rescaling. *J. Chem. Phys.* **126**, 014101 (2007).
14. S Nosé, M Klein, Constant pressure molecular dynamics for molecular systems. *Mol. Phys.* **50**, 1055–1076 (1983).
15. A Marin-Gonzalez, et al., Double-stranded RNA bending by au-tract sequences. *Nucleic Acids Res.* **48**, 12917–12928 (2020).
16. A Pérez, et al., Refinement of the amber force field for nucleic acids: improving the description of $\alpha/\gamma$ conformers. *Biophys. J.* **92**, 3817–3829 (2007).
17. J Lipfert, et al., Double-stranded RNA under force and torque: similarities to and striking differences from double-stranded DNA. *Proc. Natl. Acad. Sci. U. S. A.* **111**, 15408–15413 (2014).
18. A Marin-Gonzalez, J Vilhena, R Perez, F Moreno-Herrero, Understanding the mechanical response of double-stranded DNA and RNA under constant stretching forces using all-atom molecular dynamics. *Proc. Natl. Acad. Sci. U. S. A.* **114**, 7049–7054 (2017).
19. S Cruz-León, N Schwierz, RNA captures more cations than DNA: Insights from molecular dynamics simulations. *J. Phys. Chem. B* **126**, 8646–8654 (2022).
20. RT Young, L Czapla, ZO Wefers, BM Cohen, WK Olson, Revisiting DNA sequence-dependent deformability in high-resolution structures: effects of flanking base pairs on dinucleotide morphology and global chain configuration. *Life* **12**, 759 (2022).
21. R Stefl, et al., The solution structure of the adar2 dsrbm-RNA complex reveals a sequence-specific readout of the minor groove. *Cell* **143**, 225–237 (2010).
22. Y Huang, et al., Structural insights into mechanisms of the small RNA methyltransferase hen1. *Nature* **461**, 823–827 (2009).
23. G Masliah, P Barraud, FHT Allain, RNA recognition by double-stranded RNA binding domains: a matter of shape and sequence. *Cell. Mol. Life Sci.* **70**, 1875–1895 (2013).
24. R Lavery, M Moakher, JH Maddocks, D Petkeviciute, K Zakrzewska, Conformational analysis of nucleic acids revisited: Curves+. *Nucleic Acids Res.* **37**, 5917–5929 (2009).
25. Q Zhao, et al., Structural basis of rxr-DNA interactions. *J. Mol. Biol.* **296**, 509–520 (2000).
26. R Fernandez-Leiro, et al., The selection process of licensing a DNA mismatch for repair. *Nat. Struct. Mol. Biol.* **28**, 373–381 (2021).
27. C Zhang, et al., Twist-diameter coupling drives DNA twist changes with salt and temperature. *Sci. Adv.* **8**, eabn1384 (2022).
